# A Comprehensive Study of a New Norfloxacin-Niflumate Hydrate: Structural and Physicochemical Properties, Antibiotic Potency, Anti-Inflammatory Effect, and Drug Safety

**DOI:** 10.3390/molecules31183189

**Published:** 2026-09-10

**Authors:** Ilma Nugrahani, Yutong Wu, Sofia Fatmawati, Hidehiro Uekusa, Risang Wisesa, Masaki Uchida, Marlia Singgih Wibowo

**Affiliations:** 1 School of Pharmacy, Bandung Institute of Technology, Bandung 40132, Indonesia; fatmawatisofia@gmail.com (S.F.); risang.wisesa.utomo@gmail.com (R.W.); marlia@itb.ac.id (M.S.W.); 2 Department of Chemistry, Institute of Science Tokyo, Tokyo 152-8551, Japan; yutongwhitney@gmail.com (Y.W.); uekusa@chem.sci.isct.ac.jp (H.U.); 7654a7654@gmail.com (M.U.); 3 Faculty of Pharmacy and Science, Muhammadiyah Prof Dr Hamka University, Jakarta 13460, Indonesia

**Keywords:** norfloxacin, niflumic acid, multicomponent salt, physicochemical properties, antibiotic potency, anti-inflammation, toxicity

## Abstract

Multicomponent antibiotic–anti-inflammatory systems have recently attracted considerable attention, as this combination is becoming a standard therapy for infectious diseases. This study aimed to develop a multicomponent system comprising norfloxacin (NOR), an old fluoroquinolone antibiotic, and niflumic acid (NIF), another old, poorly soluble anti-inflammatory drug, to improve the physicochemical properties, antibiotic potency, and anti-inflammation effect, as well as their safety. First, a phase diagram was constructed to ensure solid-state reaction and to predict its stoichiometry; subsequently, the multicomponent system was prepared by solvent-drop grinding. The product was analyzed by a series of thermal analyses and powder X-ray diffraction (PXRD). Next, Fourier-transform infrared spectroscopy and nuclear magnetic resonance elucidated the molecular interactions, and the final 3D structure was determined by single-crystal X-ray diffraction, followed by Hirshfeld surface analysis. Afterward, the solubility and chemical stability were assessed using high-performance liquid chromatography, and the physical stability of the multicomponent system was evaluated by PXRD. Antimicrobial potency against Gram-negative and Gram-positive bacteria, as well as anti-inflammatory activity in vivo, were also evaluated. The results demonstrated that a newly formed antibiotic–anti-inflammatory multicomponent system, named norfloxacin–niflumate (NORNIF), in a salt dihydrate form, significantly improved the stability and antibiotic potency of NOR, including against the resistant microbe, as well as the solubility and in vivo anti-inflammatory effect of NIF simultaneously. In addition, preliminary in silico studies using Swiss-ADME and ProTox-3 predicted that the salt was well absorbed in the gastrointestinal tract and could be classified as toxicity class 4 (non-toxic).

## 1. Introduction

Norfloxacin (NOR) is a first-generation fluoroquinolone antibiotic with broad-spectrum activity that specifically inhibits bacterial DNA replication. NOR exhibits relatively low aqueous solubility, and it has been associated with neurotoxicity compared with newer-generation antibiotics [1,2,3]. In addition, the antibiotic is less stable when exposed to light due to the piperazine amine site (Figure 1a) [4,5], and its potency must be increased to overcome bacterial resistance due to the misuse of these drugs [6].

In the case of infections, antibiotics are occasionally combined with nonsteroidal anti-inflammatory drugs (NSAIDs) to alleviate inflammation and pain and to prevent disease progression [7,8]. Niflumic acid (NIF), whose structure is shown in Figure 1b, is an NSAID with low solubility [9]. One popular method to increase solubility is to form salts [10,11,12]. Our previous research showed that ionic interactions with flufenamic acid and NIF increased the solubility and antibiotic potency of the fluoroquinolone levofloxacin. On the other hand, both solubility and anti-inflammatory effects also increased. Thus, we found that the antibiotic–anti-inflammatory multicomponent salt overcame the weaknesses of each drug [13,14]. The increase in antibiotic potency may be due to the antibiotic adjuvant effect of NIF against *Staphylococcus aureus* [15]. In addition, NSAIDs are potentially neuroprotective due to their selective mechanism of action at GABA receptors [16].

This study aimed to develop a norfloxacin–niflumate (NORNIF) multicomponent system to enhance its physicochemical properties, antibiotic potency, and anti-inflammatory effects and to conduct an in silico toxicity study. Solid-state reactions and analyses were performed to reduce the use of organic solvents in accordance with green chemistry principles [17]. Firstly, the molar ratio was determined using a binary phase diagram, and then solvent-drop grinding (SDG) was used to construct the multicomponent system. The solid-state character of the product was subsequently characterized using thermal analysis, including an electrothermal apparatus to measure the melting point, differential scanning calorimetry (DSC), differential thermal analysis/thermogravimetry (DTA/TG), and powder X-ray diffraction (PXRD). The structure was determined using Fourier-transform infrared spectroscopy (FTIR), nuclear magnetic resonance (NMR), and single-crystal X-ray diffractometry (SCXRD). Furthermore, a high-performance liquid chromatography system was developed to determine the drug concentration in the solubility and stability test sample, and PXRD was also used to assess stability. Antibiotic potency against Gram-negative and Gram-positive bacteria, including a resistant strain, in vivo anti-inflammatory tests, and in silico ADME and combined toxicity predictions were also conducted to determine the impact of salt formation on the drug’s efficacy and toxicity.

## 2. Results

### 2.1. Molar Ratio Screening Result

A series of NOR-NIF molar ratios plotted against melting temperature yielded a binary phase diagram (Figure 2), indicating a solid-state multicomponent system, as evidenced by its “W”-shaped profile [18,19]. The two eutectic points were between 0.3 and 0.7 molar fractions of NOR in the mixture, which may correspond to molar ratios of NOR: NIF about 1:2, 1:1, and 2:1. Therefore, in the next step, the multicomponent system was prepared with those compositions.

### 2.2. Multicomponent System Preparation and Characterization 

The three molar ratios (1:2, 1:1, and 2:1) were mixed and processed by solvent-drop grinding (SDG) with ethanol as the solvent. This process triggered molecular interactions between NOR and NIF in the solid phase, yielding fine white powders. They were then measured using the electrothermal method to determine the melting point, which usually corresponds to the appropriate molar ratio, yielding the data in Table 1. The 1:1 molar ratio of NOR to NIF showed the lowest melting point among the proportions, suggesting that complete reaction of the multicomponent system occurred at that ratio, which should then be confirmed by PXRD. 

Next, PXRD of the multicomponent system candidates showed that the 1:1 molar ratio exhibited a completely new pattern, with no residual peaks from the individual starting drugs; meanwhile, the 2:1 and 1:2 still showed the NOR and NIF, respectively. The diffractogram for a 1:1 molar ratio is depicted in Figure 3, with the single components compared, showing distinctive peaks at 2θ = 7.0, 12.3, 13.1, 19.3, and 21.9°, which do not belong to NOR and NIF.

Next, Figure 4 presents the thermal profile of NORNIF. First, the DSC thermogram in Figure 4a shows that NORNIF (1:1) produced by SDG has a different melting point than the individual components, NOR and NIF, indicating that a new solid phase has formed. Only one broad endothermic peak was observed in the NORNIF thermogram between 110 and 136 °C, which was previously predicted to correspond to its melting point. However, based on a visual inspection of the electrothermal analysis in Figure 4b, the sample was first wetted at 110 °C, then shrank to 137 °C, and finally melted at 148–150 °C. To clarify the different thermal profiles, TG/DTA was performed, yielding the thermogram in Figure 4c. The DTA resembled the DSC profile but included an exothermic curve representing recrystallization, and the TG showed a two-step weight loss of 5.6%, corresponding to the release of two water molecules. Elaborating on thermal analysis data from three instruments, it was predicted that NORNIF was a hydrate phase that released the water in two steps from up to 136–137 °C. In the tight, closed-pan DSC, those waters released and then dissolved the crystal; hence, no other peaks were observed in the thermogram until degradation. But in the open system of electrothermal analysis and DTA/TG (the sample pan was not closed), after losing all water molecules, the sample recrystallized, then melted at 148–150 °C, and finally oxidized gradually from ~184 °C. Subsequently, the multicomponent system was strongly predicted to form a solid-state hydrate. This data was subsequently confirmed by single-crystal X-ray diffractometry in the final step of the structural study, following FTIR and NMR analyses.

### 2.3. Structural Study

Based on the ΔpKa between NOR (pKa, 8.34; nitrogen on the piperazinyl ring [20]) and NIF (pKa, 1.9; carboxylic acid [21]), which is greater than 3, the interaction between them to form a multicomponent system was predicted to be ionic, yielding a salt like that reported for NOR–phenolic acid and NOR–mefenamate [10,11] and for the other fluoroquinolone–fenamic acid multicomponent systems such as levofloxacin niflumate and levofloxacin–flufenamate [13,14].

The structural study of NORNIF in solid-state form was performed using FTIR and ^1^H-NMR. Figure 5 shows FTIR (a) and NMR (b) data. The FTIR spectra of NOR in Figure 5a (bottom) show the stretching vibration of the secondary amine (N-H) of the piperazine ring in the 3421 cm^−1^ region. This peak is relatively broad and often overlaps with the O-H stretching vibration of the carboxylic acid group. Next, the peak at 1731 cm^−1^ represents the carboxylic acid, and the band at 1623 cm^−1^ is attributed to C=O stretching of the pyridone (cyclic ketone) of that antibiotic. On the other hand, NIF spectra had the C=O and O-H stretching of carboxylic acid at 1670 and 3320 cm^−1^ [11].

As a reference, the physical mixture of NOR–NIF 1:1 (without any treatment) exhibited all characteristic peaks at 1300–1750 cm^−1^, but there was an overlap in the bending peaks in the functional group area. Meanwhile, the NORNIF multicomponent system spectra showed several changes: first, the shifting of the secondary amine peak from 3436 to 3421 cm^−1^ and a distinctive new peak at 2923 and 1581 cm^−1^, which was then predicted as the presence of the protonated secondary amine of NOR [22]; next, the presence of C-O asymmetric stretching of the NIF’s carboxylic acid, as the counterion, was represented by the appearance of new bands at 1504 cm^−1^. The other peaks in those areas were considered evidence of a change in vibrational energy following the new interaction; i.e., the NOR spectrum at 1731 cm^−1^ was predicted to shift to 1712 cm^−1^ due to stretching of newly formed hydrogen bonds in its carboxylic acid. Subsequently, it was confirmed that the amine of NOR interacted ionically with the carboxylate of NIF. Finally, the peaks between 2500 and 3500 cm^−1^ can be attributed to hydrogen bonding of water, forming the hydrate state of NORNIF, consistent with thermogram data.

Next, ^1^H-NMR analysis was conducted to confirm the ionic interaction between NOR and NIF in solution, with results shown in Figure 5b, with tetramethylsilane (TMS) as an internal standard. NOR (black line) and NORNIF (blue line) showed a triplet for the methyl protons (-CH_3_) at δ = 0.76–0.85 ppm and a quartet for the methylene protons (-N-CH_2_-) at δ = 4.11–4.47 ppm. For methylene groups that are attached to the piperazine ring, a doublet was observed at δ = 3.17–3.27. Aromatic protons for NOR, NIF (red line), and NORNIF were observed around δ = 6.74–8.42. NOR showed the hydrogen that bonds with the amine piperazine at δ = 8.64 ppm, and the hydrogen of the carboxylic acid of NIF is represented by a peak at δ = 10.18 ppm. Meanwhile, the NORNIF multicomponent system showed a shift to a higher-field peak (δ = 8.67) for the proton in the piperazine group of NOR. This peak shift might imply that the hydrogen atom of the piperazine moiety in NOR experienced a different chemical environment. On the other hand, the absence of the carboxylic acid resonance in NORNIF, formerly observed at δ = 10.18 ppm in NIF, suggests that deprotonation of this functional group has occurred, interacted with the counterion, and formed a salt. As noted, the intense peak at δ = 7.23 in NOR, NIF, and NORNIF was attributable to the solvent residue CDCl_3_ [23]. Thus, these H-NMR data helped to confirm that NORNIF was a salt, a finding subsequently corroborated by a 3D structural study using SCXRD.

### 2.4. 3D Structure Determination Using SCXRD

SCXRD supported the final confirmation of the structure of the multicomponent system. The 3D structure data are depicted in Figure 6. Figure 6a shows the salt molecular structure with two water molecules. Next, Figure 6b shows the crystal lattice in the triclinic system with space group P-1 (No. 2), revealing two pairs of NORNIF units per unit cell. Figure 6c depicts two water molecules included in the crystal, both refined with an occupancy of 1.0. Two hydrogen residual electron density peaks were observed near the N32 atom of NOR in the difference Fourier map, indicating that N32 was protonated. In addition, the difference between the two C-O bond lengths in the carbonyl group of NIF is 0.028 Å (<0.03 Å), suggesting that it existed as a carboxylate. The observations indicated that the NOR-NIF crystal is a salt dihydrate. A void with a volume of 32.15 Å^3^ was calculated around the center of the unit cell using Mercury [24]; the probe radius was 1.2 Å, and the grid spacing was approximately 0.3 Å, computed using the “contact surface” option. However, no residual electron density was observed in this void, indicating that no solvent molecules are present within it. Upon verifying the resulting structure, the calculated diffractogram matched the measured data, as shown in Figure 6d. Hereafter, the 3D structure of the multicomponent system was proven to be the NORNIF dihydrate salt, with the summarized crystal data listed in Table 2 and Supplementary File S1.

Furthermore, Hirshfeld surface mapping was performed to visualize, confirm, and quantify the noncovalent interactions in the multicomponent system, with the results shown in Figure 7. Prominent red spots on the Hirshfeld surface mapping of NORNIF in Figure 7a underscore the ionic intermolecular bond (O^−^...N-H^+^) driving the salt formation [25]. Next, the interactions were mirrored in the 2D fingerprint plots (Figure 7b), which illustrated the percentage contribution of each interaction to the total d_norm_. The result revealed that H...O/O...H and H...H (van der Waals forces) interactions were the dominant forces: each accounted for 29% of the total contacts, as visualized by sharp bottom spikes for H...O/O...H and a broad blue area for H...H. Non-covalent F...H bonds also contributed to the NORNIF intermolecular interaction at 18%, followed by C...H bonds at 13%, and pi-pi stacking interactions between the aromatic rings, indicated by C...C bonds, at 4%. On the other hand, the sharp bottom spike for N...H/H...N accounted for about 3% of the d_norm_ surface.

### 2.5. Solubility Experimental Data

Solubility data in Figure 8a and Table 3 showed that NOR was less soluble in water (0.194 ± 0.003 mg/mL) compared to NOR as NORNIF dihydrate salt (0.048 ± 0.008 mg/mL). On the other hand, the solubility of NIF in water increased ~2-fold, from 0.059 ± 0.001 mg/mL to 0.129 ± 0.003 mg/mL as the NORNIF salt form. 

The solubility of NOR alone (0.361 ± 0.003 mg/mL) was higher in phosphate buffer of pH 6.8 compared to in water but still showed a 3-fold decrease as NORNIF salt (0.102 ± 0.001 mg/mL). For NIF solubility, a 1.4-fold increase was observed as NORNIF salt (0.100 ± 0.004 mg/mL) compared to NIF alone (0.073 ± 0.004 mg/mL).

### 2.6. Stability Experimental Data

Following salt formation, a modified stability study was conducted to evaluate the resulting physicochemical properties. The stability study was conducted over 4 weeks under actual ambient conditions in Bandung, Indonesia, where humidity and UV intensity are generally higher than those specified under standard conditions [26,27,28]. The results of the stability test are shown in Figure 9. The NORNIF color did not visually change up to week 4 in an open container (observed: 22–30 °C/70–85% RH), whereas NOR and NIF turned yellow after the first week (Figure 9a). Also, the diffractogram of the salt remained stable, whereas NOR changed over the 2θ range of 10–30° (Figure 9b). The concentration of NOR was assessed using the validated HPLC method (Supplementary File S2), and it was found that the concentration of single NOR decreased by 14.14% in the final week.

### 2.7. Antibiotic Potency Improvement

The NORNIF (1:1) dihydrate salt showed greater antibiotic potency against Gram-negative bacteria, e.g., *Escherichia coli* (MIC 0.016 μg/mL) and *Salmonella typhi* (MIC 0.031 μg/mL), with ~2 and ~4-fold increases relative to NOR alone (0.031 μg/mL for *E. coli* and 0.125 μg/mL for *S. typhi*), respectively (Figure 10).

Next, the salt also showed greater activity against Gram-positive bacteria, *Staphylococcus aureus* (MIC 0.157 μg/mL) and *Methicillin-resistant Staphylococcus aureus* (MRSA) (MIC 3.125 μg/mL), with ~2-fold increases relative to single NOR (0.313 μg/mL for *S. aureus* and 6.25 μg/mL for MRSA), respectively (Figure 10). NIF alone did not provide a clear solution in the microplate at concentrations > 25 μg/mL. In addition, data showed that both single drugs and NORNIF were more potent against Gram-negative than Gram-positive bacteria.

### 2.8. In Vivo Anti-Inflammatory Performance

NORNIF dihydrate salt exhibited a higher anti-inflammatory effect than its single NIF and NORNIF physical mixture (Figure 11). A 10–20% reduction in inflammation was observed with NORNIF dihydrate salt relative to the control from 30 min to 360 min after carrageenan induction. A significant difference (asterisk, *p*-value < 0.05) in anti-inflammatory effects between the NORNIF salt and NIF alone was observed by a post hoc Tukey test at the early stage of inflammation, between 30 min and 120 min.

### 2.9. In Silico ADME and Safety Study

The BOILED-Egg and ADME predictions from SwissADME are presented in Figure 12 and Table 4. The red dots in the BOILED-Egg in Figure 12a, shown by NIF and NORNIF salt, reveal no efflux from the central nervous system by the P-glycoprotein (PGP-) substrate. NOR with the blue dots indicates that the P-glycoprotein (PGP+) substrate effluxed the compound. Originally, NOR is in HIA (human intestinal absorption) and NIF in BBB (blood–brain barrier) permeability, but the NORNIF salt was in the grey area. Meanwhile, NORNIF salt parameters on the bioavailability radar (Figure 12b) fall within the pink area, indicating that these substances are orally bioavailable. Interestingly, the ProTox-3 toxicity predictions in Table 4 indicated lower toxicity of NORNIF salt (LD50: 4336 mg/kg; toxicity class 4) than the single NOR (LD50: 1000 mg/kg; toxicity class 4) and NIF (LD50: 250 mg/kg; toxicity class 3) [29]. Moreover, based on the in silico prediction data in Table 4, NORNIF salt can also be expected to reduce the risk of a single compound’s neurotoxicity, nephrotoxicity, and hepatotoxicity.

## 3. Discussion

A multicomponent phase, such as a co-crystal or salt, is likely to be generated when the binary phase diagram shows a “W” profile, as this pattern indicates the potential presence of strong molecular interactions, ionic bonding characteristic of salt formation, or, at least, hydrogen bonding capable of promoting co-crystal formation [19]. Based on the binary phase diagram in Figure 2, the NOR and NIF showed the potential to form a multicomponent system with stoichiometric balance between the two eutectic points at 0.3 and 0.7 molar fractions of NOR, or equal to 2:1, 1:1, and 2:1, respectively [29]. 

However, Table 1 shows that only the 1:1 molar ratio has the narrowest melting point range, and the PXRD in Figure 3 confirmed that only the 1:1 molar ratio was fixed. This indicates that only one site of each compound interacted and suggests that this site was the secondary amine in the piperazine ring of NOR (pKa: 8.51), which conferred higher basicity, while the most acidic site of NIF (pKa: 1.9) was the carboxylic acid. Thus, the calculation yielded a scale distance of more than 3 pKa units between the two drugs, prompting salt formation via ionic interaction. 

Next, the broad endothermic peak in the thermogram of NORNIF in Figure 4, consistent with electrothermal and DTA/TG observations, indicated water molecule release upon heating or hydrate formation. In the closed-pan DSC measurement, the water release overlapped with the melting point range of 110–137 °C (Figure 4a). Also, the thermal profile was confirmed by visual observation, using the electrothermal method in Figure 4b, which showed that NORNIF became wet at 110 °C, shrank up to 137 °C, and melted at 148–150 °C.

After that, DTA/TG, which used an open-pan sample as usual, facilitated water evaporation during heating. The elaborated DTA thermogram showed a profile similar to the DSC result, but it exhibited an exothermic recrystallization curve after hydrate release. Meanwhile, thermogravimetry showed a weight loss in two steps: from the starting point to 110 °C and then from 110 °C to 137 °C, totaling 5.6% and corresponding to the loss of two water molecules (Figure 4c). These findings indicate that two distinct molecular-weight-reduction energies indicate a dihydrate form. Hereafter, these results support the conclusion that a new solid-state hydrate phase has formed via the NOR-NIF (1:1) reaction.

Regarding the starting structures in Figure 1, NOR contains several synthons, including fluor, ketone, and the hydroxy group of a carboxylic acid, as well as a secondary amine. Meanwhile, NIF also has fluor, a secondary amine, in addition to the carboxylate, which had ionically interacted with the NOR’s secondary amine. After the formation of the multicomponent system, those sites became more polar, and it was easier for them to interact with water molecules. Solid-state molecules can interact at relatively low energy, such as through hydrogen or ionic bonding [30,31].

Next, the hypothesized structure was successfully elucidated by FTIR, NMR, and SCXRD. FTIR spectra in Figure 5a indicated interaction between amine and carboxylic acid. The stretching vibration of the amine piperazine of NOR and the carbonyl of the carboxylic acid of NIF were lost, but new peaks at 1504, 1581, and 2923 cm^−1^ appeared, indicating that an ionic interaction has occurred. Also, the peaks between 2500 and 3500 cm^−1^ indicated neutral hydrogen bonding. FTIR analysis confirmed the presence of a salt hydrate multicomponent system, consistent with the thermogram profile.

The NMR spectra in Figure 5b were used to analyze ionic interactions in solution. CDCl_3_ was a good solvent for the analysis of organic salts (drugs) because it could dissolve drug compounds and had a low dielectric constant, meaning it lacked the strength required to dissociate the anion and cation of the salts being measured. Protons that transferred due to salt formation, which is a proton from NIF’s carboxylic acid, formerly observed at δ = 10.21 ppm, would experience extreme deshielding after forming a salt. The chemical shift in neighboring protons of NOR’s piperazine moiety also shifted from 3.13 to 3.27 ppm [32]. This observation is in line with previous reports on the NOR reaction with other anthranilic acid derivatives such as mefenamic acid [10] and flufenamic acid [33]. That finding also met with the salt formation theory, which states that it needs a pKa distance of more than 3 [34], whereas the pKa of NOR is 8.51 at the piperazine amine; meanwhile, the carboxylic acid moiety of NIF has a pKa of 1.9. Unfortunately, the NMR spectra of NOR, NIF, and NORNIF salt did not perform well due to the low resolution of the NMR instrument. Therefore, the structural analysis continued using SCXRD to determine the arrangement of atoms within the crystal.

Finally, the structural study was confirmed by SCXRD, and the results in Figure 6 a–c clearly show the positions of ionic interactions and the hydrogen bonding of water molecules, as well as the crystal lattice system, with the data listed in Table 1. Meanwhile, the diffractogram, also shown in Figure 6d, confirmed the accuracy of the crystal structure determination. A notable intermolecular interaction was a pair of charge-assisted hydrogen bonds. One was between the -NH^+^ piperazine of NOR and the -COO^−^ moiety of NIF, and the other was NH^+^…O=C-OH (NOR). Two water molecules were observed within the NORNIF unit, confirming that NORNIF was a dihydrate salt. Both water molecules bridged two NIF anions by OH…O^−^ or OH…N hydrogen bonds. NOR formed an intramolecular hydrogen bond (OH…O) and donated two intermolecular hydrogen bonds to the water molecules. Hydrogen bonds play a significant role in this conformation, with intramolecular and intermolecular interactions within NOR, NIF, and water molecules providing the structural geometry and stabilizing the crystal. The electronic cloud of interaction has also been confirmed by Hirshfeld surface analysis, which revealed all non-covalent bindings (Figure 7a) and detailed their respective percentages in Figure 7b. Crystal structure data can be obtained free of charge with the submission number CCDC 2500551, which contains the supplementary crystallographic data for this paper, from the Cambridge Crystallographic Data Center at www.ccdc.cam.ac.uk/structures (accessed on 7 May 2026).

The new thermodynamic entity of a multicomponent salt with a distinctive 3D arrangement certainly affects the compound’s physicochemical characteristics such as solubility and stability, which, in turn, influence the drug’s performance. From the experiment, NIF’s solubility increased significantly, as shown in Figure 8, like the previous salts reported: norfloxacin–mefenamate [10] and enoxacin–niflumate [12]. The salt form facilitated its dissociation in water, changed its polarity, and increased its crystal surface area. The solubility improvement might also be due to proton transfer between molecules within the hydrophilic regions of the salt’s crystalline structure. On the other hand, like other fluoroquinolones, NOR’s solubility depended on pH due to its amphoteric structure, which comprised the amine–piperazine and carboxylic acid moieties. The decrease in NOR’s solubility might be due to the intermolecular interaction structure of the NORNIF crystal. Hydrophobic interactions such as π-π stacking and C-F may affect the crystal system’s affinity for the solvent [35].

Based on the data, the saturated NORNIF solution in water had a pH of 5.8, lower than that of the single NOR solution (6.8; Table 3). References indicate that NOR has maximum solubility at pH 6.8–7.0 [36]. The solubility test was also performed in phosphate buffer of pH 6.8 and showed a higher solubility value for NOR. Due to the greater abundance of neutral molecular species over the cationic form caused by the carboxylic acid group, decreased solubility was observed in vitro. The other fluoroquinolones (ciprofloxacin and levofloxacin), in the form of cocrystals or salts, have been reported to exhibit different dissolution profiles across several pH values due to their zwitterionic nature [37,38]. However, in the gastrointestinal tract, the buffering capacity of physiological fluids may not be significantly affected by the drug due to dynamic volume renewal, unlike in laboratory dissolution or in vitro solubility experiments; therefore, it was assumed that the final pH solution might affect the rate of absorption, but the bioavailability of NOR as NORNIF salt requires further research [36,37,39].

Next, the stability might change physically and chemically. In general, the fluoroquinolones exhibit pseudo-polymorphism under humid conditions, including NOR. NOR can form anhydrous, dihydrate, trihydrate, hemipentahydrate, and pentahydrate polymorphs [40]. In addition, oxidative degradation converts it into a potent phototoxic compound [5]. Both physical and chemical instability reduce purity, thereby affecting antibiotic potency. In this study, a four-week stability test was intentionally conducted under actual ambient conditions in Bandung, Indonesia, where the average relative humidity exceeds 70%, and cumulative light exposure reaches approximately 4 million lux-hours [26]. These environmental conditions are considerably more stressful than the ICH-recommended stability conditions of 65% relative humidity and 1.2 million lux-h [27,28], providing a more stringent assessment of the solid-state stability of the prepared system. Such conditions were considered particularly relevant for evaluating the material’s susceptibility to moisture- and light-induced transformations, which are critical factors affecting the physicochemical integrity of pharmaceutical solids. Moreover, the use of open containers was intended to maximize direct interaction between the samples and the surrounding environment, thereby facilitating detection of hydrate formation, phase transformations, and photostability under UV exposure. This approach was particularly justified for fluoroquinolone compounds, which are known to be susceptible to photodegradation and environmental stressors [41]. Further research might be conducted on the stability of fluoroquinolones under more extreme controlled conditions to better elucidate their degradation processes and mechanisms.

The instability characteristic of NOR was evidenced by a color change after a week of storage (Figure 9a) and by changes in its diffractogram (Figure 9b), which were confirmed to be those of a pentahydrate [40]. Hydrate formation altered the molecular weight, thereby decreasing the active compound content, with the NOR concentration decreasing to 85.86%. Meanwhile, the solid state of the multicomponent salt was more resistant to humidity due to its more compact crystalline structure with several types of non-covalent intermolecular interactions (Figure 7b), as evidenced by the unchanged diffractogram in Figure 9b [42]. Briefly, the formation of the NORNIF multicomponent salt improved its chemical and physical stability.

Furthermore, the change in polarity was predicted to affect the drug interaction with the microbial membrane. Figure 10 shows that NOR and its salt, NORNIF, exhibited lower MIC values, indicating greater effectiveness in *E. coli*. That phenomenon may be due to their mechanism of action, which targets DNA gyrase in Gram-negative bacteria and porins in their outer membranes, thereby facilitating antibiotic entry and increasing intracellular antibiotic concentrations. Meanwhile, Gram-positive bacteria exhibited higher MICs to NOR than Gram-negative bacteria because they have thicker peptidoglycan layers and efflux pump mechanisms that reduce intracellular antibiotic concentrations [43]. Next, the increase in NORNIF’s antibiotic potency relative to NOR is predicted to result from changes in polarity and the lipid–water partition coefficient. It was established that NOR acted as a concentration-dependent killing (CDK) antibiotic, with its bactericidal activity increasing with increasing concentration relative to the bacterial MIC [6]. Based on the SwissADME calculation, the XLOGP3 of NOR increased upon conversion to the salt form, from −1.03 to 2.66; this change is presumed to increase its transport across bacterial cell membranes with multiple layers of lipopolysaccharide in Gram-negative bacteria or peptidoglycan in Gram-positive bacteria [12]. NIF is also reported as an antibiotic adjuvant [44]; therefore, it is assumed that NIF can help maintain the concentration of NOR within bacterial cells within its effective range. Further tests, such as a checkerboard assay and time-kill assay, are needed to better elucidate how the mechanism of drug–drug salt formation influences the antibiotic potency [45].

The increase in NSAID solubility enhances the drug concentration in the blood, hence improving the anti-inflammatory effect in vivo, as shown in Figure 11. The salt’s anti-inflammatory activity in acute Wistar rat paw inflammation was higher than that of NIF alone at the early stage of inflammation. Equal concentrations of NOR and NIF, alone or in mixture, served as controlled variables. At these equivalent concentrations, the lowest inflammatory response was observed with the NORNIF salt compared with the control, single NIF, or the physical mixture of NOR and NIF. This suggests that NIF in the form of NORNIF salt exhibits enhanced anti-inflammatory activity compared to its single form. This beneficial activity can be attributed to solubility, as discussed before, and to a change in the lipid–water partition coefficient, resulting in a faster onset of action. In the experiment, carrageenan induced acute paw inflammation in Wistar rats, resulting in a biphasic edema response. The initial phase, typically manifesting within the first hour post-injection, was driven by the secretion of inflammatory mediators, including serotonin, bradykinin, and histamine. Then, the late phase was characterized by neutrophil infiltration into the injured tissue, the COX-2-mediated synthesis of prostaglandins from arachidonic acid, and the emission of proinflammatory cytokines, specifically TNF-α and IL-1β [46]. As is well known, the solubility parameter plays a significant role in the bioavailability and effects of NSAIDs, particularly in their mechanisms of action, as they reach the peripheral nervous system [47]. Specifically, NOR showed a higher percentage of inflammation than the control at 180 min, consistent with its known side effects, including allergic and tendon injuries, such as swelling or pain [48]. In addition, the delayed effect observed after administration of NORNIF was predicted to result from NOR’s slower absorption from the gastrointestinal tract. In brief, NORNIF might reduce inflammation and side effects associated with NOR.

Finally, Figure 12 showed the predictive ADME study results. The Brain or Intestinal Estimated Permeation (BOILED-Egg) method, a SwissADME feature that simultaneously predicts passive gastrointestinal absorption (HIA) and brain access (BBB), was used (Figure 12a). That model was based on two physicochemical parameters: WLOGP and TPSA (topological polar surface area). Changes in the salt molecule area were driven by changes in WLOGP and TPSA, which underlie the BOILED-Egg model. On the other hand, the lipophilicity of NIF became lower after reacting with NOR. The ionic bond that occurs between the piperazine group of NOR and the carboxylate acid group of NIF causes a change in polarity, as reported in NIF salts with levofloxacin and enoxacin [12,13].

Next, one of the outputs of the SwissADME web tool, which predicts lipophilicity, dimensions, polarity, insolubility, unsaturation, and flexibility, is called the bioavailability radar. The pink region in Figure 12b depicts the ideal range for those six parameters. Lipophilicity for raw material and NORNIF salt, determined by XLOGP3, ranges from −7 to +5 [49]. The solubility result of NOR in the form of the NORNIF salt decreased in line with the SwissADME calculations, which indicate an increase in XLOGP3 and a decrease in LOG S (ESOL) (Table 4). NORNIF salt’s molecular weight (MW) exceeded 500 g/mol and violated the Lipinski rule (Table 4). However, the salt would dissociate back into NOR and NIF in the digestive tract. Additional characteristics for the NORNIF salt include polarity (TPSA) of more than 140 Å and solubility (logS) [50] which does not exceed 6. 

In fact, fluoroquinolones were reported to have a risk of toxicity to the nervous system and CNS when administered together with NSAIDs [51]. The suspected mechanism of toxicity is the presence of NSAIDs, which affect their interaction with the same receptor, namely the gamma-aminobutyric acid-A (GABA A) receptor. The R7 side chain, unsubstituted 7-piperazine, plays an important role in the fluoroquinolone toxicity pathway. The formation of salts through interaction between NOR’s piperazine and NIF’s carboxylic acid may offer additional potential to reduce the toxicity of both drugs [51]. The toxicity class and LD50 of the salt using ProTox-3 (Table 4) showed a predicted lower risk of toxicity compared to the single drug. Predictions of organ toxicity in the kidneys, neuro, and liver also decreased, which was likely caused by changes in polarity and its interaction with receptors due to the salt’s unique pharmacokinetic and receptor affinity properties [52]. Further in vitro and in vivo toxicity testing is required to demonstrate the toxicity-reducing effect observed in the salt.

Hereafter, based on the experimental data, the salt derived from NOR with NIF can be further developed into a solid dosage form such as a tablet or capsule. However, further studies, including in vivo toxicity and pharmacokinetic assessments, should be conducted to ensure the safety and efficacy of this new antibiotic–anti-inflammatory salt multicomponent system.

## 4. Materials and Methods

### 4.1. Chemicals and Materials

Active pharmaceutical ingredients NOR 98%, NIF 98%, and λ-carrageenan were obtained from TCI (Tokyo, Japan). Analytical-grade ethanol, sodium carboxymethyl cellulose (CMC Na), KBr for FTIR, CDCl_3_ for ^1^H NMR characterization, sodium dihydrogen phosphate, methanol, and triethylamine for HPLC assay were purchased from Sigma-Aldrich (Jakarta, Indonesia). For the antibiotic potency test, nutrient agar and Mueller–Hinton Broth from HiMedia (Maharashtra, India) were used as growth media. Gram-negative bacteria *Escherichia coli* (ATCC 8939) and *Salmonella typhi* (ATCC 14029), and also Gram-positive bacteria Staphylococcus aureus (ATCC 6538) and *Methicillin-resistant Staphylococcus aureus* (ATCC 33591), were cultured in the Microbial Laboratory at Bandung Institute of Technology (Bandung, Indonesia). Distilled water was provided by the Bandung Institute of Technology (Bandung, Indonesia). Meanwhile, for in vivo testing, male Wistar rats were obtained from CV Kencana (Bandung, Indonesia).

### 4.2. Stoichiometric Screening and Preparation of the NORNIF Multicomponent System

A total of 100 mg of powder consisting of NOR and NIF was mixed homogeneously at varying molar ratios (0:10, 1:9, 2:8, …, 10:0). Molar ratio screening was performed by constructing a binary phase diagram and plotting the melting temperatures of the physical mixture as a function of molar composition. Commonly, a “W” profile with two eutectic points may predict the new solid-state reaction (salt or cocrystal) with the stoichiometric proportion within those temperatures [53].

Based on that screening, the salt was prepared using solvent-drop grinding (SDG) with ethanol. A 100 mg physical mixture powder of the predicted NORNIF molar ratios (1:2, 1:1, and 2:1) was ground in a 10 cm diameter marble mortar, then 1 mL of 98% ethanol was added, and the mixture was ground homogeneously until it became wet. The solvent was then evaporated at room temperature, yielding a dry powder. SDG products with a narrow melting range were then subjected to further characterization. 

All solid samples, including raw materials, physical mixtures, and reaction products, were analyzed using solid-state analytical techniques: melting point analysis, DSC, DTA/TG, and PXRD for solid-phase analysis, followed by FTIR, NMR, and SCXRD for structure determination.

### 4.3. Solid-State Characterization

#### 4.3.1. Melting Point Analysis Using the Electrothermal Method

A 3–5 mg powder sample was placed in a melting point capillary tube, inserted into an Electrothermal AZ 9003 apparatus from Cole-Palmer (Staffordshire, UK), and heated to the appropriate temperature at a heating rate of 10 °C/min. Its melting point was determined by observing it through the device’s magnifying glass.

#### 4.3.2. DSC 

Approximately 1–3 mg of the sample was ground, placed in an aluminum pan for DSC (Rigaku, Tokyo, Japan), sealed with a press tool, and inserted into the sample holder of a DSC Thermoplus EVO2-8231 (Rigaku, Tokyo, Japan). An empty aluminum pan was placed next to the sample pan as a reference, and the instrument was routinely calibrated using indium to ensure the validity of the measurement. The measurement was performed over a temperature range of 30–250 °C at a heating rate of 10 °C/min. The measurement was done twice per sample.

#### 4.3.3. DTA/TG

The test was carried out using a DTA/TG Hitachi STA7300 (Ibaraki, Japan). 3–5 mg of NORNIF salt powder was weighed and placed in an aluminum pan. The pan containing the sample and an empty pan as a reference were placed in the sample holder. Measurements were taken with nitrogen gas at a flow rate of 50 mL/min and a heating rate of 10 °C/min. The measurement was carried out twice per sample.

#### 4.3.4. PXRD 

Approximately 100 mg of the sample was evenly spread into the sample holder of a Rigaku SmartLab X-ray diffractometer (Rigaku, Tokyo, Japan) using Cu Kα radiation at 40 kV and 15 mA. Measurements were performed over a 2θ range of 3–50° at a scan speed of 10°/min using the D/teX Ultra2 detector with a divergence slit of 1.25° and an incident height-limiting of 10 mm. The measurement was performed twice per sample. OriginPro^®^ 2024 software composed the diffractogram [54].

### 4.4. Structure Determination

#### 4.4.1. FTIR Analysis

1–2 mg of each sample was homogeneously mixed with anhydrous KBr at a 1:100 ratio, spread onto a plate, and compacted into transparent KBr pellets. Measurements were taken over the wavenumber range of 4000–400 cm^−1^ using an FTIR 4200 type A from Jasco (Tokyo, Japan) at a speed of 2 mm/s and a resolution of 16 cm^−1^. The measurement was performed twice per sample. OriginPro^®^ 2024 software was used to compose the spectra.

#### 4.4.2. ^1^H NMR Analysis

Samples of 5 mg were dissolved in 1 mL of CDCl_3_ and placed in 5 mm NMR tubes, with tetramethylsilane (TMS) added as an internal standard. The clear solution was then analyzed using a Benchtop NMR-60 Spectrometer from Nanalysis Scientific Corporation (Calgary, Alberta, Canada) at room temperature (25 ± 5 °C) with an operating frequency of 60 MHz under a relaxation delay of 1.93 s, a resolution of 0.179 Hz/pt, a spectral range of −0.2–13 ppm, and a spectral width of 735.29 Hz. The data were processed using Nanalysis NMReady v2.0.7 software (Calgary, AB, Canada). OriginPro^®^ 2024 software was used to compose the spectra.

#### 4.4.3. SCXRD 

A colorless single crystal (0.170 × 0.140 × 0.110 mm) obtained from solvent evaporation using an ethanol–water (9:1) mixture was used for the SCXRD study. The diffraction measurement was performed using a Rigaku XtaLAB Synergy R DW system (Tokyo, Japan) with a HyPix X-ray detector and CuKα-focused X-rays (Tokyo, Japan). The initial crystal structure was determined using SHELXT-2019/3 [55]. The positions of hydrogen atoms were calculated geometrically and included in the least-squares refinement using the riding atom model. Different Fourier maps identified several important hydrogen atoms bonded to water molecules and nitrogen atoms, and their positions were refined.

To evaluate the intermolecular interactions within the crystal lattice, the NORNIF 3D structure was analyzed via CrystalExplorer 21.5 to generate Hirshfeld surfaces and 2D fingerprint plots. This approach provided a quantitative breakdown of how different interaction types influence the overall packing arrangement and the crystal’s stability. 

### 4.5. HPLC Validation Method and Solubility Test

First, a quantitative reverse-phase HPLC method was validated for solubility and stability testing. The instrument an HPLC e2695 from Waters (Milford, MA, USA) is equipped with a 2489 UV–visible detector from Waters (Milford, MA, USA) to determine the concentration of chemical compounds for the solubility test. The stationary phase utilized was a Zorbax Eclipse XDB-C18 column (4.5 × 250 mm, 5 µm) from Agilent (Santa Clara, CA, USA). The phosphate buffer (25 mM) was prepared by mixing 1.7 g of KH_2_PO_4_ with 400 mL of bi-distilled water and 0.5 mL of triethylamine, and then adjusting the pH to 2.7. The solution was diluted to 500 mL with bi-distilled water in a volumetric flask. 

The mobile phase was prepared by mixing 150 mL of methanol with 350 mL of the phosphate buffer solution at pH 2.7. The optimal mobile phase was a 75:25 (*v*/*v*) mixture of methanol (p.a.) and phosphate buffer (25 mM, pH 2.7) in isocratic mode for the analysis of NOR, NIF, and NORNIF. Calibration curves were plotted based on six standard concentrations, both as individual standards and as mixtures. To create the mixed calibration curve, the highest concentration of each standard was combined and then diluted to yield six concentrations. This solution was shaken, filtered through a 0.2 µm filter membrane, and sonicated for 15 min. A 10 µL sample injection volume and a flow rate of 1 mL/min were used. All analyses were conducted in triplicate. The UV detector was set to 274 nm for the NOR and 285 nm for the NIF. The validation parameters assessed include accuracy, precision, linearity, and measurement range. The NOR and NIF concentrations in the stability and solubility tests will subsequently be determined using this HPLC-validated method. 

For the solubility test, 100 mg of powder was weighed and mixed with 10 mL of distilled water (pH 7.0) and phosphate buffer (pH 6.8) in Erlenmeyer flasks; each sample was prepared in triplicate. Sonicator-assisted mixing was performed at room temperature (25 ± 3 °C) for 3 h to ensure complete saturation, as indicated by the existence of remaining insoluble particles and an unchanged concentration during measurement. To determine the solution’s pH, a Mettler Toledo pH meter (Küsnacht, Switzerland) was used. The saturated solution was then filtered using a 0.22 µm membrane filter. The filtered solution was diluted and analyzed by HPLC using a validated method, with measurements repeated 3 times for each sample. 

### 4.6. Stability Test

The stability procedure was conducted over a four-week period (short-term stability) under room conditions in Bandung, Indonesia. These local environmental conditions result in higher humidity and UV exposure than those specified in the ICH stability testing standard [27,28]. The primary aim was to assess the alterations that arise when comparing NORNIF with its constituent components (NOR and NIF). An amount of 150 mg of sample powder was evenly distributed on an open Petri dish, subsequently stored in an open area at ambient conditions in Bandung, Indonesia (75 ± 5% RH/25 ± 3 °C), monitored using a thermohygrometer (Taffware, Indonesia) [26], and exposed to uncontrolled sunlight. The color and PXRD profiles of the samples were monitored weekly, and the sample concentrations were measured in the first and final weeks [54].

### 4.7. Antibiotic Potency Test

An antibiotic potency test was performed using a partially quantitative microdilution method. The medium used was Mueller–Hinton broth (MHB), which was poured into a 96-well microplate. *Escherichia coli* (ATCC 8939), *Salmonella typhi* (ATCC 14029), Staphylococcus aureus (ATCC 6538), and *Methicillin-resistant Staphylococcus aureus* (ATCC 33591) were used in this research. Bacterial suspensions were prepared from bacterial cultures incubated at 37 ± 0.2 °C for 24 h in MHB and then diluted to a concentration of 0.5 McFarland (1.5 × 10^8^ CFU/mL). The final concentration was prepared by diluting the bacterial suspension in MHB to 10^6^ CFU/mL. Total plate counts were performed on nutrient agar to quantify microbial cell count.

Next, the microdilution method, performed in a 96-well microplate, consists of 12 columns and 8 rows. Each sample (NOR and NORNIF) was dissolved in pH 7.0 water at a concentration equivalent to the NOR content (2, 10, and 25 μg/mL). After that, 100 µL of MHB was introduced as a negative control in column 1. For the positive control, column 2 was filled with 100 µL of MHB and 10 µL of the bacterial suspension. The other wells were filled with 100 µL of MHB. 

Subsequently, each sample was diluted to half-concentration in MHB. These 100 µL sample solutions were added to column 12 (highest concentration). In column 11, 100 µL of MHB was combined with 100 µL of the solution from column 12. This dilution process was performed sequentially from column 11 to column 3 with a final well volume of 100 µL. Lastly, 10 µL of the diluted bacterial suspension was added to wells in columns 3–12. The microplate was sealed, labeled, and incubated at 37 °C for 24 h. Minimum inhibitory concentration (MIC) was defined as the lowest concentration at which the samples remained clear in the microplate after incubation, indicating no microbial growth. This microdilution potency test was performed in triplicate for each sample [42].

### 4.8. In Vivo Anti-Inflammatory Test

Firstly, the Health Research Ethics Committee of the School of Pharmacy, ITB, granted ethical clearance for the animal study (KEPHP/2025/VII/130725IK/TSBK). The drug samples were prepared by making an oral suspension solution using CMC Na 0.9%. Anti-inflammatory testing was conducted in 25 male Wistar rats (age, 2–3 months, with body weight of 150–200 g) randomly divided into five groups of 5 rats: negative control (CMC Na 0.9%), positive control (NIF 22.5 mg/kg BW), NOR (26.2 mg/kg BW), a physical mixture of NOR and NIF, and NORNIF salt, which was equivalent to the NIF dosage. Before the test, the rats were acclimated to adequate water and food for 7 days inside polypropylene cages (62 cm × 40 cm × 20 cm) with a stainless-steel wire cover under ambient conditions in Bandung, Indonesia (24 ± 2 °C). 

Subplantar edema was induced by injecting 0.1 mL of 1% λ-carrageenan into the left paw of the rat 30 min after oral administration of the suspension samples in 0.9% CMC-Na, with a maximum oral volume of 2 mL/200 g BW. A plethysmometer measured the volume of inflammation of the rat’s paw before and at 30, 60, 120, 180, 240, 300, and 360 min following carrageenan induction. After the test, the rats were treated in accordance with ethical guidelines. The degree of inflammation was calculated as the percentage difference between initial edema and edema after therapy. Data were presented as means and standard deviations in Microsoft Excel. Statistical analysis, including normality tests (Shapiro–Wilk and Kolmogorov–Smirnov; *p*-value > 0.05), one-way ANOVA, and Tukey post hoc tests (*p*-value < 0.05), was performed using GraphPad Prism 11 software to draw conclusions [56]. 

### 4.9. In Silico ADME and Toxicity Prediction

NOR, NIF, and NORNIF salt structure models were created using ChemDraw 22.2 and converted to “Simplified Molecular Input Line Entry System” (SMILES) code. Predictions of physicochemical properties, lipophilicity, water solubility, pharmacokinetics, and druglikeness were generated using the SwissADME web service from the Swiss Institute of Bioinformatics (http://www.swissadme.ch/ accessed on 7 May 2026). Toxicity predictions were made using the ProTox-3 web service (https://tox.charite.de/protox3/ accessed on 7 May 2026) by entering SMILES codes and selecting the model prediction option (organ toxicity and toxicity endpoints) [57]. The calculations were performed in June–July. 

## 5. Conclusions

In simple terms, NOR reacted with NIF to form a solid, multicomponent system, the NORNIF dihydrate salt. The salt improved the stability of both components and the solubility of NIF. The salt’s antibiotic potency against *E. coli*, *S. typhi*, *S. aureus*, and MRSA also indicates that the antibiotic–anti-inflammatory multicomponent system enhanced NOR’s potency. Moreover, NORNIF showed improved in vivo anti-inflammatory effects. Furthermore, preliminary ADME and in silico toxicity studies predicted improved gastrointestinal absorption of the salt and reduced neurotoxicity, nephrotoxicity, and hepatotoxicity, thereby reducing NIF from three to four toxicity classes. Hereafter, this new antibiotic–NSAID multicomponent salt can be further developed, supported by several studies on pharmaceutics, pharmacokinetics, and safety regulatory considerations.

## Figures and Tables

**Figure 1:**
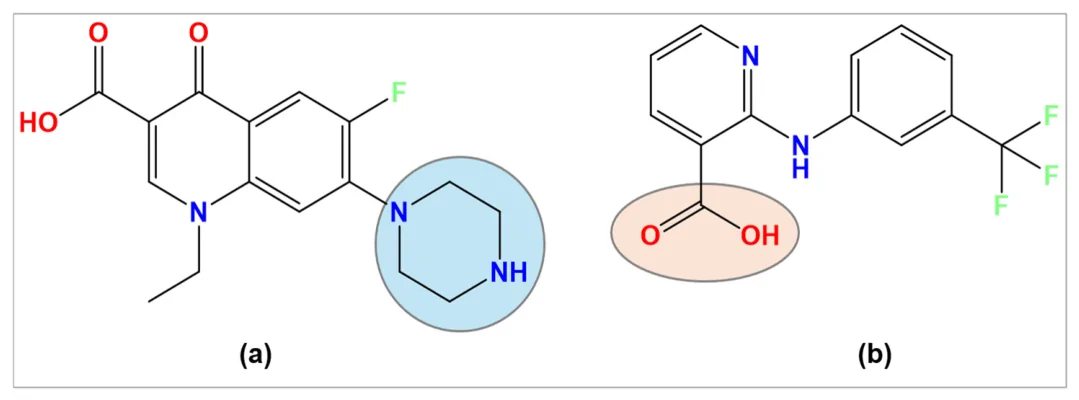
The structure and predicted interaction site of (**a**) norfloxacin and (**b**) niflumic acid.

**Figure 2:**
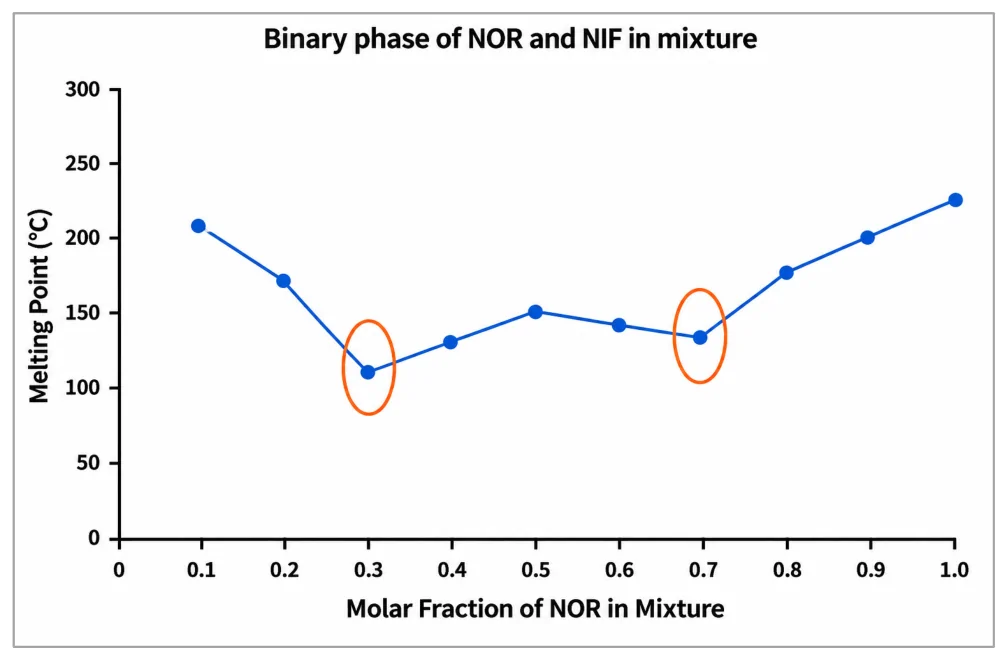
Binary phase diagram of NOR and NIF mixture with an area between two eutectic points at 0.3 and 0.7 molar fraction of NOR.

**Figure 3:**
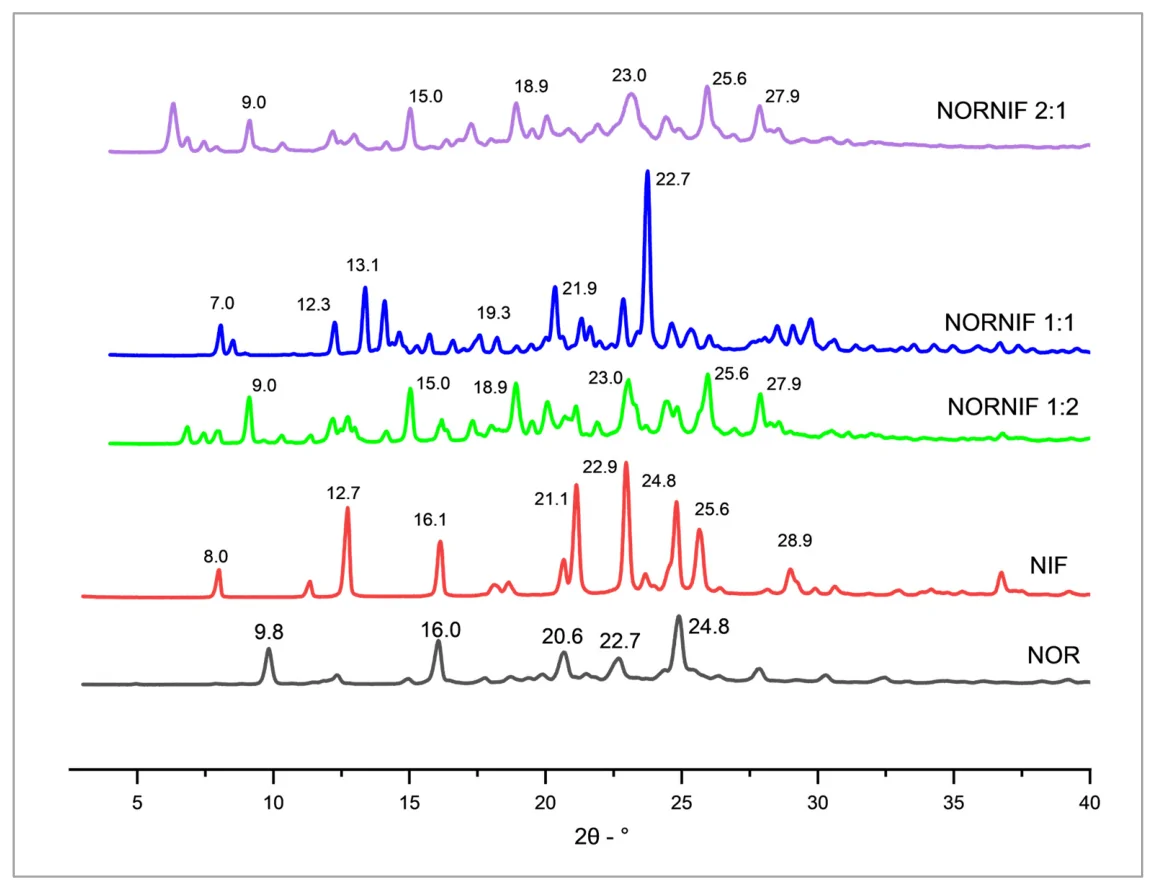
PXRD diffractogram of NOR, NIF, and NORNIF 1:2, 1:1, and 2:1 multicomponent systems.

**Figure 4:**
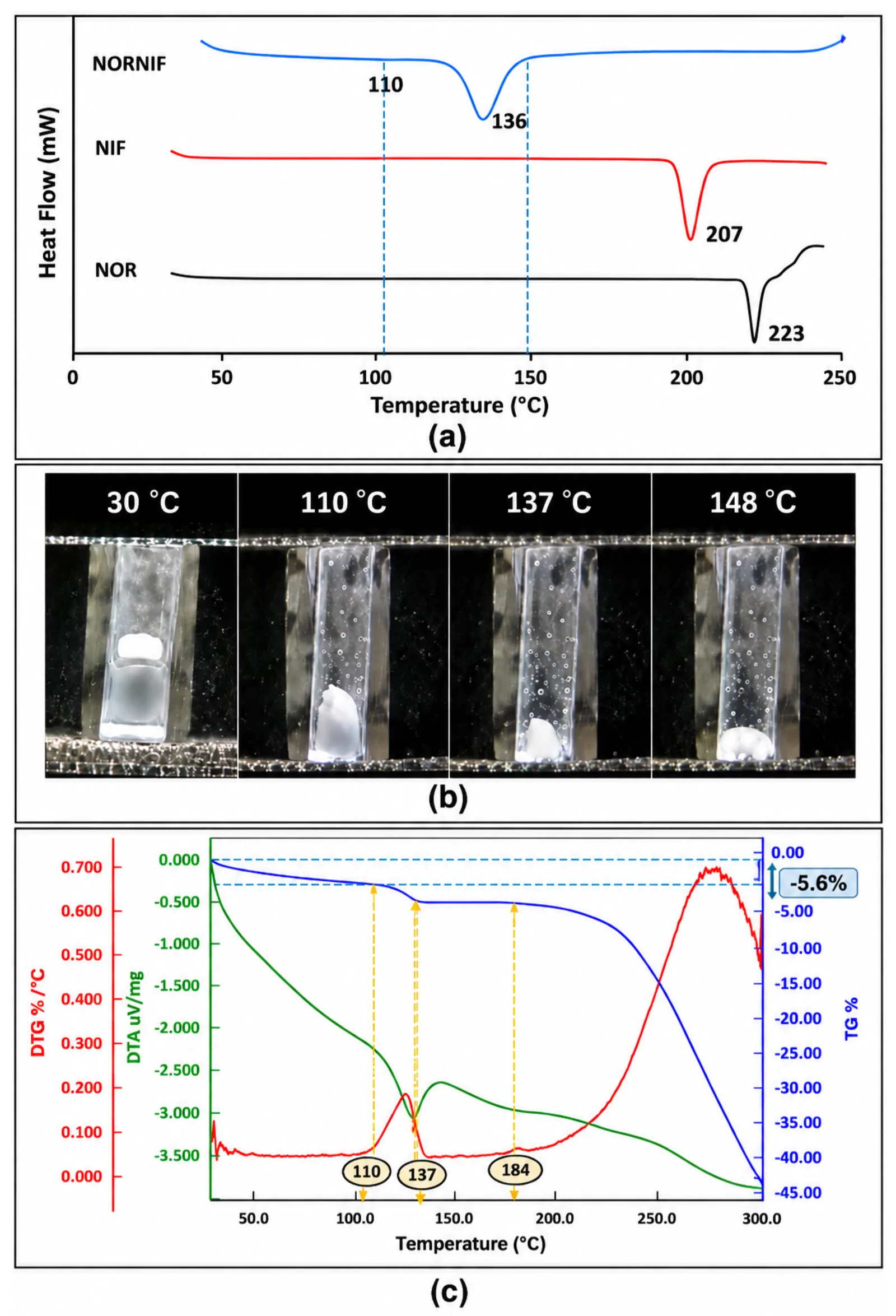
(**a**) DSC curve of NOR, NIF, and NORNIF multicomponent system, (**b**) melting point observation of NORNIF using an electrothermal melting point apparatus, (**c**) TG/DTA thermogram of NORNIF.

**Figure 5:**
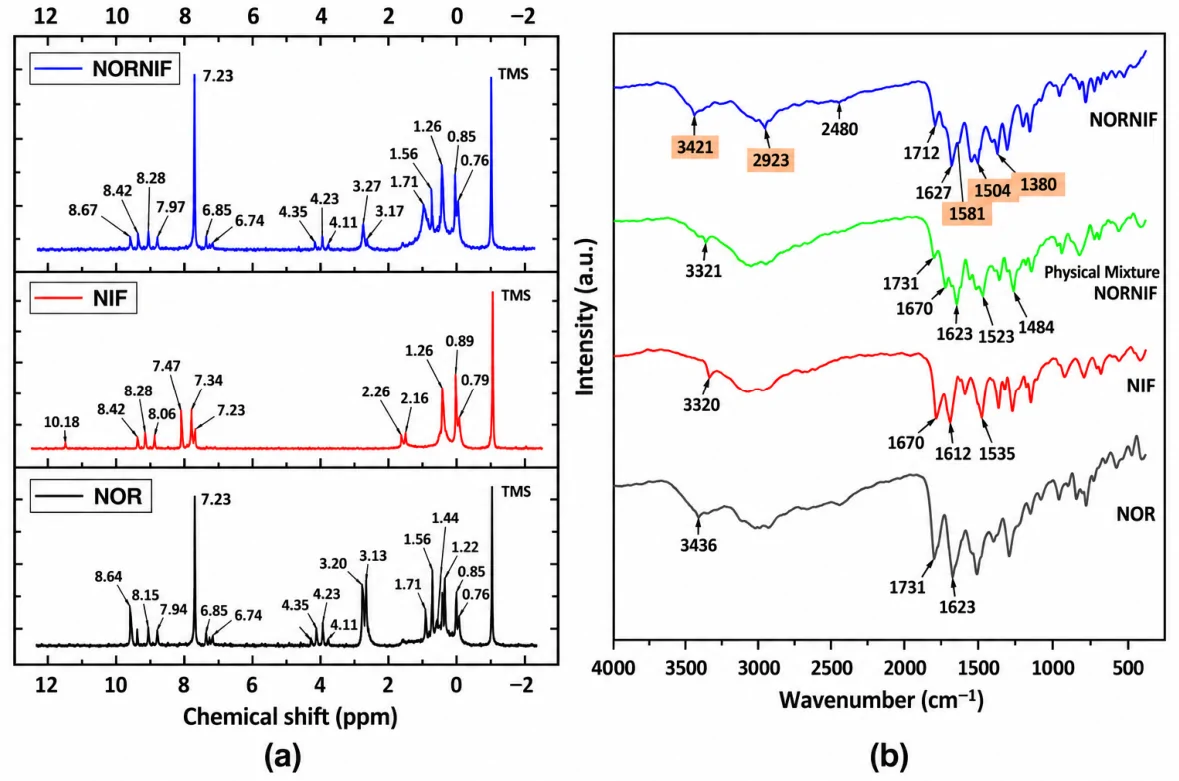
(**a**) FTIR spectra of raw materials, NORNIF physical mixture, and NORNIF multicomponent system. The new bands are depicted in the orange marks; (**b**) ^1^H-NMR spectra of raw materials and NORNIF multicomponent system in CDCl_3_ solution.

**Figure 6:**
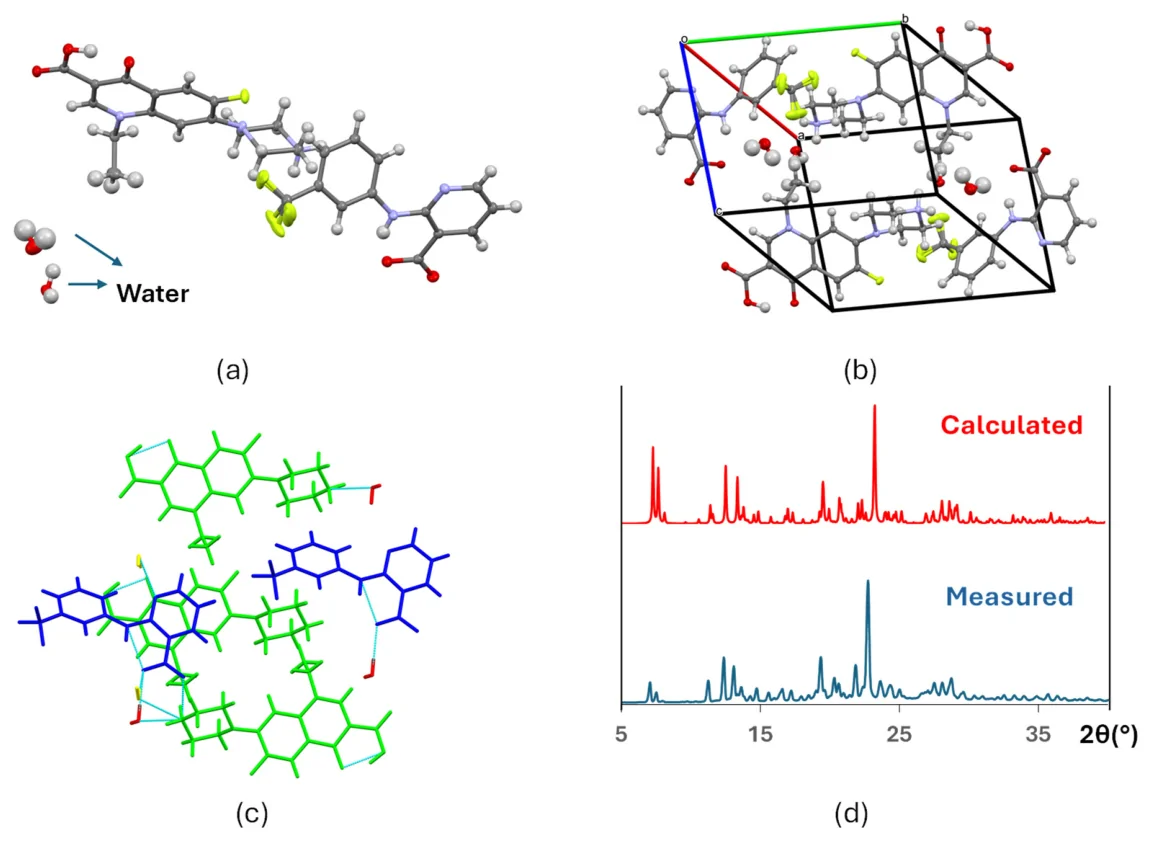
(**a**) Salt molecular structure, (**b**) packing structure viewed on the ab plane, (**c**) molecular stacking diagram viewed along [−1 1 1] direction with the sea-blue lines being hydrogen bondings, (**d**) comparison between calculated (upper) and measured (lower) PXRD of NORNIF dihydrate salt.

**Figure 7:**
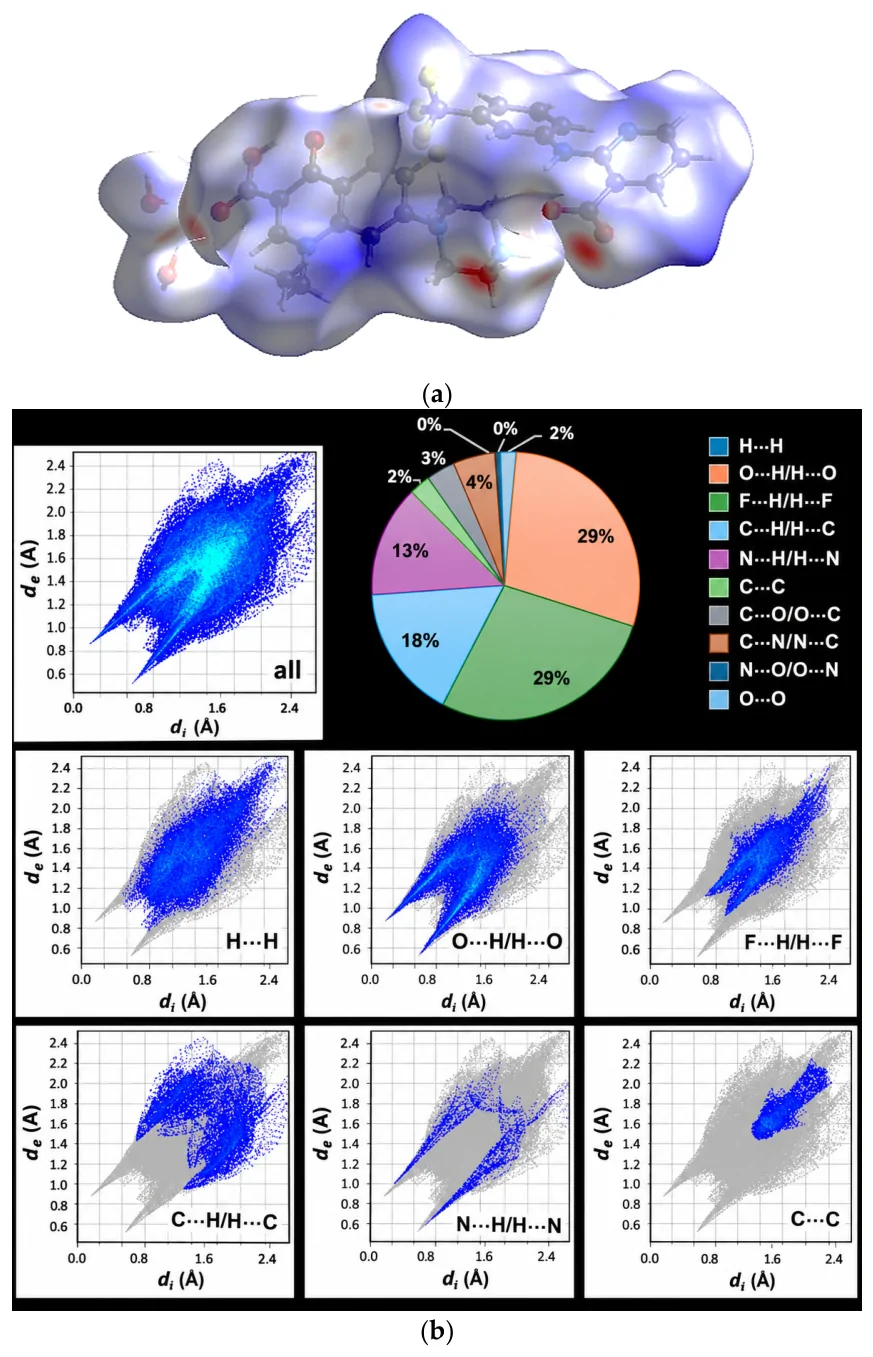
(**a**) Hirshfeld surface analysis of NORNIF salt, (**b**) 2D fingerprint plots for NORNIF with a pie chart showing the percentage of intermolecular interactions.

**Figure 8:**
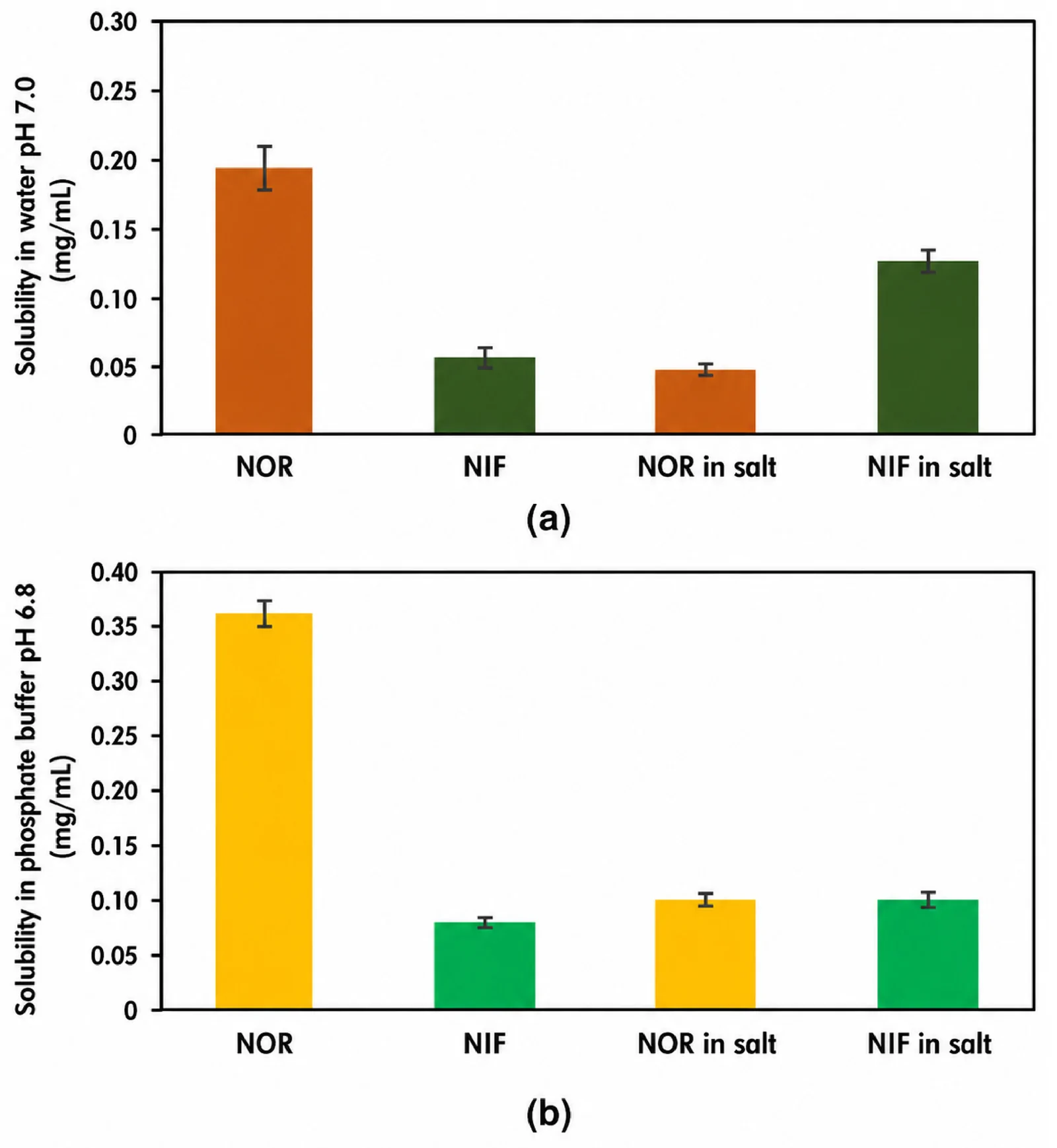
Solubility data of NORNIF compared to the single components (**a**) in water of pH 7.0 and (**b**) in phosphate buffer of pH 6.8 (*n* = 3).

**Figure 9:**
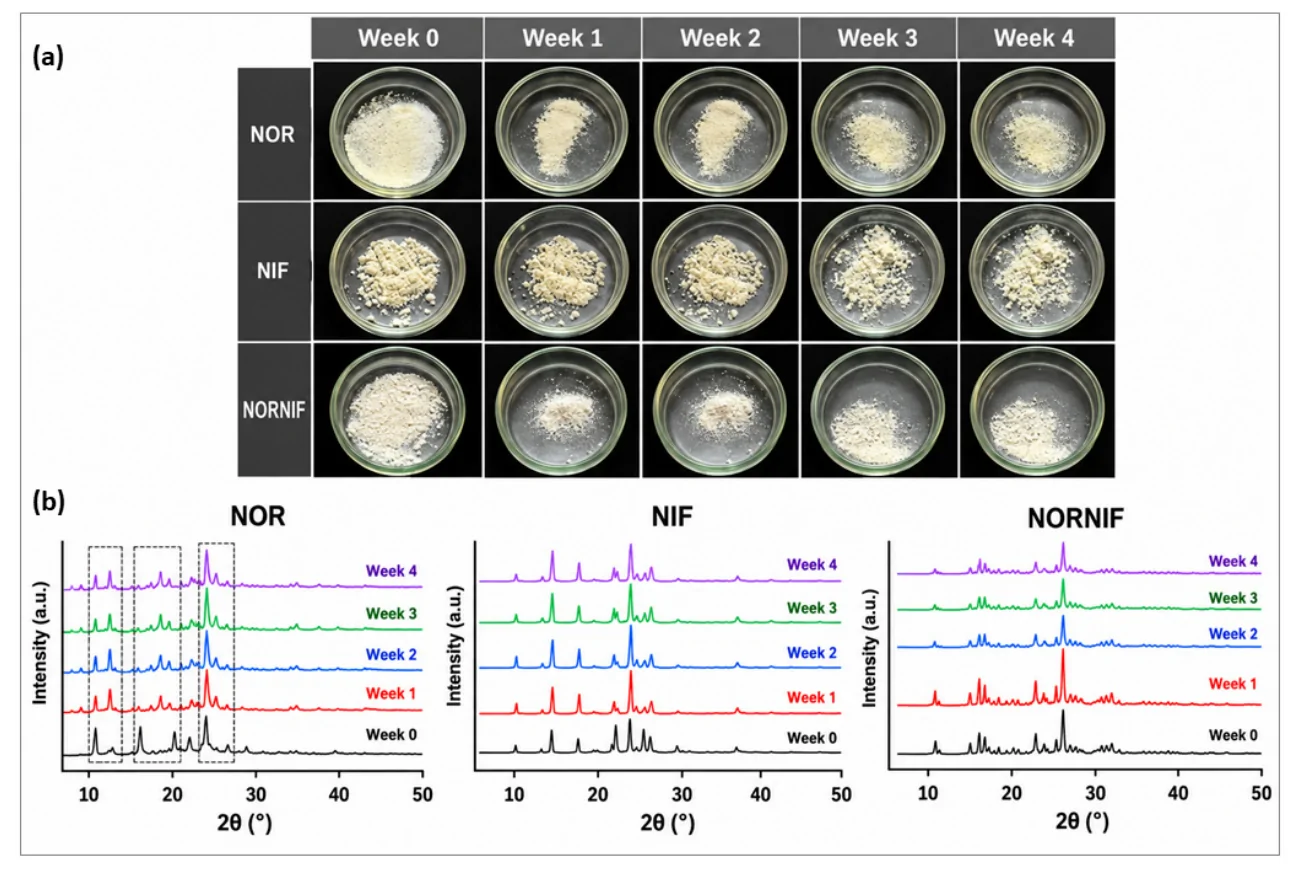
Stability test result of NORNIF dihydrate salt and raw material for 3 weeks: (**a**) visual color; (**b**) PXRD profile.

**Figure 10:**
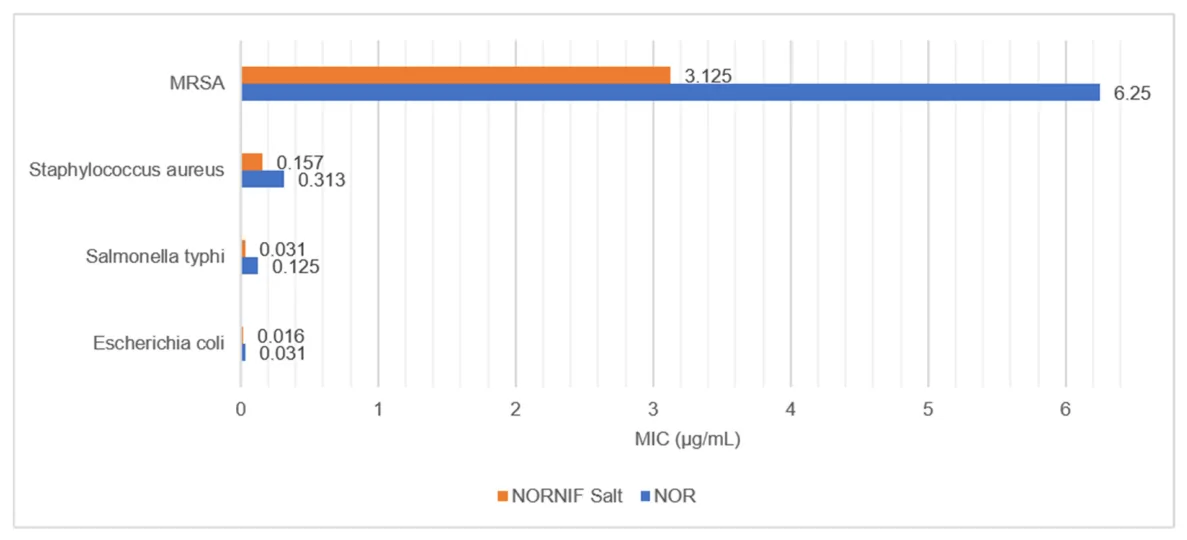
Minimum inhibitory concentration of NOR (blue) and NORNIF (1:1) salt (orange) against *Escherichia coli, Salmonella typhi*, *Staphylococcus aureus*, and MRSA (*n* = 3).

**Figure 11:**
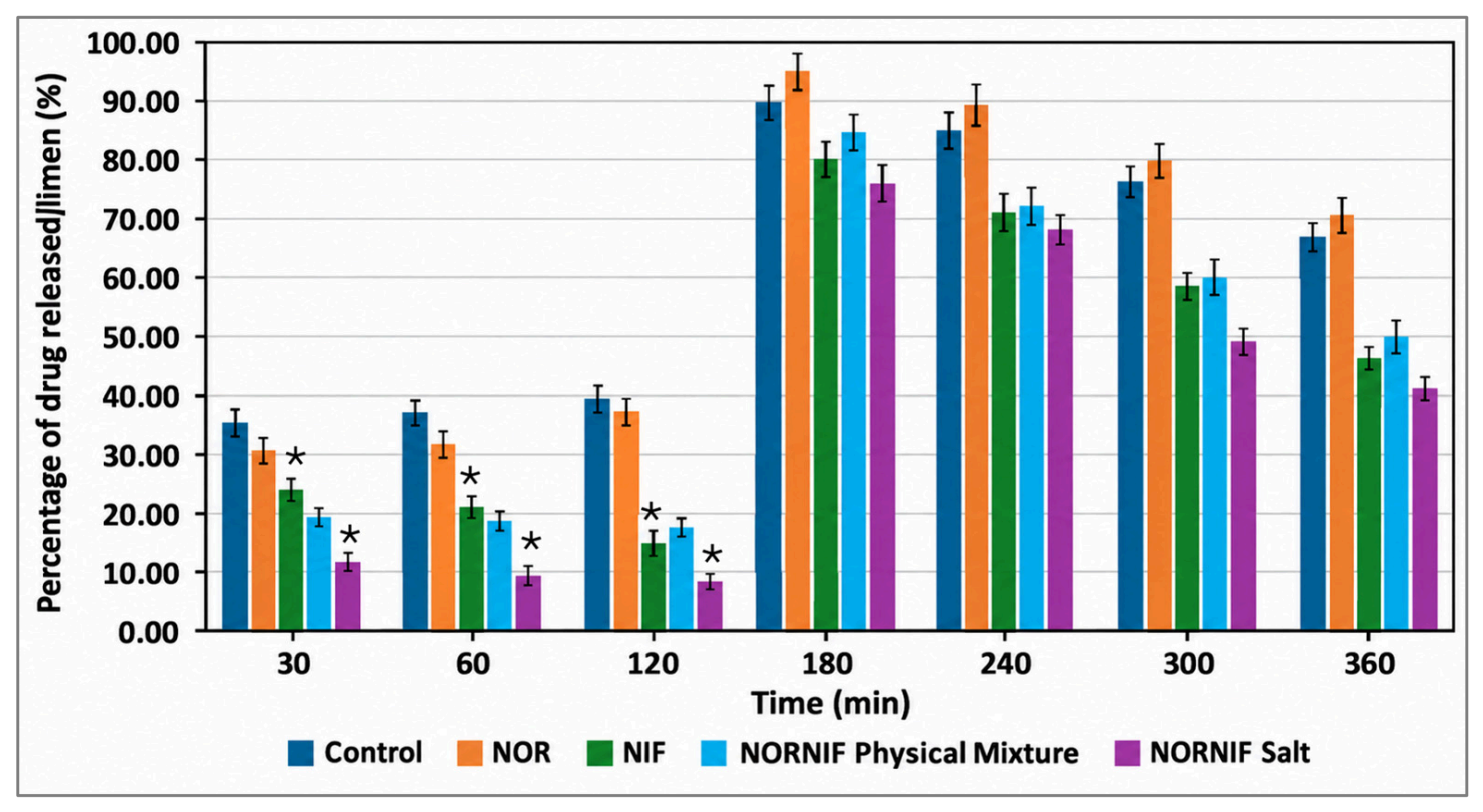
The anti-inflammatory performance of the control, NOR, NIF, NORNIF physical mixture, and NORNIF salt is shown as the percentage of inflammation (*n* = 5). The (*) signs show the statistically significant difference based on the post hoc Tukey test (*p* < 0.05).

**Figure 12:**
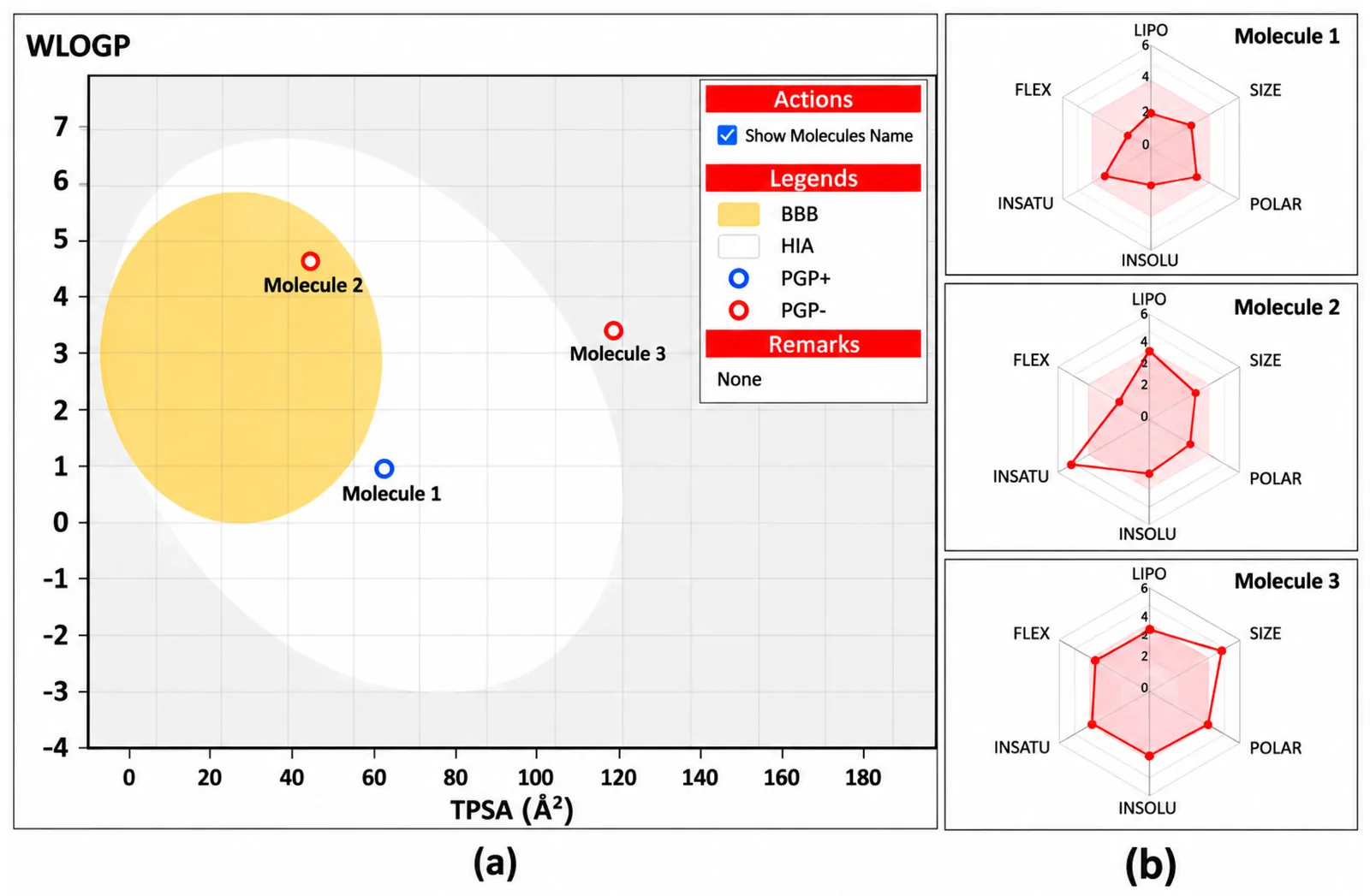
ADME prediction result from SwissADME tools: (**a**) BOILED-Egg diagram for the NOR (Molecule 1), NIF (Molecule 2), and NORNIF (Molecule 3); (**b**) bioavailability radar for NOR, NIF, and NORNIF.

**Table 1:** Melting range determination of NOR, NIF, and NORNIF using the electrothermal melting point apparatus.

No	Compound	Melting Range (°C)
1	NOR	221–224
2	NIF	205–208
3	NORNIF (1:2) SDG	110–118
4	NORNIF (1:1) SDG	148–149
5	NORNIF (2:1) SDG	170–178

**Table 2:** Crystal data of NORNIF dihydrate salt multicomponent system.

Name	Norfloxacin–Niflumate Dihydrate
Empirical formula	C_29_H_31_F_4_N_5_O_7_
Formula weight	637.59
Temperature (K)	100(2)
Wavelength (Å)	1.54184
Crystal system	Triclinic
Space group	*P*-1 (No. 2)
Cell length (Å)	*a* = 9.5148(2) *b* = 13.0829(3) *c* = 13.3165(4)
Cell angle *a* (°)	66.058(3)
Cell angle *β* (°)	88.044(2)
Cell angle γ (°)	75.078(2)
Cell volume (${Å}^{3}$)	1459.28(7)
*Z*	2
Density (g/cm^3^)	1.451
Crystal size (mm^3^)	0.170 × 0.140 × 0.110
Independent reflection	5735 [*R*(int) = 0.0537]
Goodness-of-fit on *F*^2^	1.054
Final *R* indices [*I* > 2*σ*(*I*)]	*R*_1_ = 0.0397, w*R*_2_ = 0.1131
Largest diff. peak and hole (Å^−3^)	0.246 and −0.660 e
CCDC deposition number	2500551

**Table 3:** Solubility data of NOR, NIF, and NORNIF in water.

Sample	Water (pH, 7.0)		Phosphate Buffer (pH, 6.8)	
	Solubility * (mg/mL)	Final pH	Solubility * (mg/mL)	Final pH
NOR	0.194 ± 0.003	6.8	0.361 ± 0.003	6.8
NIF	0.059 ± 0.001	4.5	0.073 ± 0.004	6.7
NOR in NORNIF	0.048 ± 0.008	5.8	0.102 ± 0.001	6.7
NIF in NORNIF	0.129 ± 0.003	5.8	0.100 ± 0.004	6.7

* Mean of triplicates ± SD.

**Table 4:** ADME and toxicity in silico analysis for NOR, NIF, and NORNIF salt using SwissADME and ProTox-3.

Parameter	NOR	NIF	NORNIF
Physicochemical Properties from SwissADME			
Molecular weight	319.33	282.22	601.55
Number of rotatable bonds	3	4	7
F.Csp ^a^	0.38	0.08	0.24
TPSA ^b^	74.57	62.22	144.20
XLOGP3 ^c^	−1.03	4.43	2.66
LOG S (ESOL) ^d^	−1.29	−4.56	−5.16
**Pharmakokinetics**			
GI absorption	High	High	Moderate
BBB permeant	No	Yes	No
P-gp substrate	Yes	No	No
CYP1A2 inhibitor	No	No	No
CYP2C9 inhibitor	No	No	No
CYP2D6 inhibitor	No	No	No
CYP3A4 inhibitor	No	No	No
**Druglikeness**			
Lipinski	Yes, 0 violations	Yes, 0 violations	Yes, 1 violation
Bioavailability score	0.55	0.85	0.56
**Toxicity Prediction from ProTox-3**			
LD50	1000	250	4336
Toxicity class	4	3	4
LogP	1.66	3.62	4.16
Hepatotoxicity	Inactive; 0.75	Active; 0.69	Inactive; 0.67
Neurotoxicity	Active; 0.98	Active; 0.63	Active; 0.84
Nephrotoxicity	Active; 0.95	Inactive; 0.52	Active; 0.80
Respiratory toxicity	Active; 0.85	Active; 0.79	Active; 0.87
Cardiotoxicity	Inactive; 0.82	Inactive; 0.72	Inactive; 0.83
Clinical toxicity	Active; 0.83	Active; 0.59	Active; 0.65

^a^ Ratio of sp3 hybridized carbons over the total amount of carbons in the molecule. ^b^ Topological polar surface area. ^c^ Parameter for lipophilicity calculations. ^d^ Estimated solubility.

## Data Availability

All data are described in the main text, figures, and supplements.

## Supplementary Materials

The following supporting information can be downloaded at: https://www.mdpi.com/1420-3049/31/18/3189/s1, CCDC 2500551 contains the supplementary crystallographic data for this paper. A summary of the crystal structure data is attached to Supplementary File S1. The data can be obtained free of charge from the Cambridge Crystallographic Data Center at www.ccdc.cam.ac.uk/structures (accessed on 7 May 2026). The chromatograms and HPLC validation results are attached in Supplementary File S2.

## Abbreviations

The following abbreviations are used in this manuscript:

ATCC	American Type Culture Collection
DSC	Differential scanning calorimetry
FTIR	Fourier-transform infrared spectroscopy
HPLC	High-performance liquid chromatography
MHB	Mueller–Hinton broth
MRSA	Methicillin-resistant *Staphylococcus aureus*
NIF	Niflumic acid
NOR	Norfloxacin
NORNIF	Norfloxacin–niflumate salt
PXRD	Powder X-ray diffraction
SCXRD	Single-crystal X-ray diffraction
SDG	Solvent-drop grinding
