# Supplementary material for: A Comprehensive Study of a New Norfloxacin-Niflumate Hydrate: Structural and Physicochemical Properties, Antibiotic Potency, Anti-Inflammatory Effect, and Drug Safety
Source: Molecules. 2026 Sep 10;31(18):3189. doi: 10.3390/molecules31183189 (PMC13635407; doi:10.3390/molecules31183189)

checkCIF/PLATON report

Structure factors have been supplied for datablock(s) nor-nif-2W_autored_a

THIS REPORT IS FOR GUIDANCE ONLY. IF USED AS PART OF A REVIEW PROCEDURE FOR PUBLICATION, IT SHOULD NOT REPLACE THE EXPERTISE OF AN EXPERIENCED CRYSTALLOGRAPHIC REFEREE.

No syntax errors found. CIF dictionary Interpreting this report

Datablock: nor-nif-2W_autored_a

Bond precision: C-C = 0.0020 A Wavelength=1.54184

Cell:	a=9.5148(2)	b=13.0829(3)	c=13.3165(4)
	alpha=66.058(3)	beta=88.044(2)	gamma=75.078(2)
Temperature:	100 K		

	Calculated	Reported
Volume	1459.28(7)	1459.28(7)
Space group	P -1	P -1
Hall group	-P 1	-P 1
Moiety formula	C16 H19 F N3 O3, C13 H8 F3 N2 O2, 2(H2 O)	C16 H19 F N3 O3, C13 H8 F3 N2 O2, 2(H2 O)
Sum formula	C29 H31 F4 N5 O7	C29 H31 F4 N5 O7
Mr	637.59	637.59
Dx,g cm-3	1.451	1.451
Z	2	2
Mu (mm-1)	1.043	1.043
F000	664.0	664.0
F000'	666.52	
h,k,lmax	11,16,16	11,16,16
Nref	5998	5735
Tmin,Tmax	0.839,0.892	0.881,1.000
Tmin'	0.838	

Correction method= # Reported T Limits: Tmin=0.881 Tmax=1.000
AbsCorr = MULTI-SCAN

```
Data completeness= 0.956          Theta(max)= 74.845

                                                 wR2(reflections)=
R(reflections)= 0.0397( 5162)                    0.1165( 5735)

S = 1.054                 Npar= 452
```

The following ALERTS were generated. Each ALERT has the format
test-name_ALERT_alert-type_alert-level.
Click on the hyperlinks for more details of the test.

Alert level C

```
PLAT601_ALERT_2_C Unit-Cell Contains Solvent Accessible VOIDS .LE.          38 Ang**3
PLAT976_ALERT_2_C Check Calcd Resid. Dens.  0.42Ang From O44      .      -0.66 eA-3
PLAT976_ALERT_2_C Check Calcd Resid. Dens.  0.54Ang From O44      .      -0.64 eA-3
PLAT977_ALERT_2_C Check Negative Difference Density on H1A        .      -0.38 eA-3
```

Alert level G

```
PLAT002_ALERT_2_G Number of Distance or Angle Restraints on AtSite          6 Note
PLAT007_ALERT_5_G Number of Unrefined Donor-H Atoms .............          2 Report
              H3      H4
PLAT172_ALERT_4_G The CIF-Embedded .res File Contains DFIX Records          1 Report
PLAT173_ALERT_4_G The CIF-Embedded .res File Contains DANG Records          1 Report
PLAT242_ALERT_2_G Low    'MainMol' Ueq as Compared to Neighbors of        C29 Check
PLAT302_ALERT_4_G Anion/Solvent/Minor-Residue Disorder (Resd    2)        15% Note
PLAT432_ALERT_2_G Short Inter X...Y Contact  O37      ..C14      .       2.97 Ang.
                                                x,-1+y,z  =      1_545 Check
PLAT480_ALERT_4_G Long H...A H-Bond Reported H13B     ..F48      .       2.60 Ang.
PLAT480_ALERT_4_G Long H...A H-Bond Reported H20      ..F48      .       2.63 Ang.
PLAT860_ALERT_3_G Number of Least-Squares Restraints ............          6 Note
PLAT912_ALERT_4_G Missing # of FCF Reflections Above STh/L=  0.600        261 Note
PLAT969_ALERT_5_G The 'Henn et al.' R-Factor-gap value ..........      3.900 Note
              Predicted wR2: Based on SigI**2  2.99 or SHELX Weight 11.05
PLAT978_ALERT_2_G Number C-C Bonds with Positive Residual Density.         16 Info
```

---

0 **ALERT level A** = Most likely a serious problem - resolve or explain
0 **ALERT level B** = A potentially serious problem, consider carefully
4 **ALERT level C** = Check. Ensure it is not caused by an omission or oversight
13 **ALERT level G** = General information/check it is not something unexpected

0 ALERT type 1 CIF construction/syntax error, inconsistent or missing data
8 ALERT type 2 Indicator that the structure model may be wrong or deficient
1 ALERT type 3 Indicator that the structure quality may be low
6 ALERT type 4 Improvement, methodology, query or suggestion
2 ALERT type 5 Informative message, check

It is advisable to attempt to resolve as many as possible of the alerts in all categories. Often the minor alerts point to easily fixed oversights, errors and omissions in your CIF or refinement strategy, so attention to these fine details can be worthwhile. It is up to the individual to critically assess their own results and, if necessary, seek expert advice.

PLATON version of 26/09/2025; check.def file version of 20/09/2025

---

duplicate check

No duplication found

Datablock nor-nif-2W_autored_a - ellipsoid plot

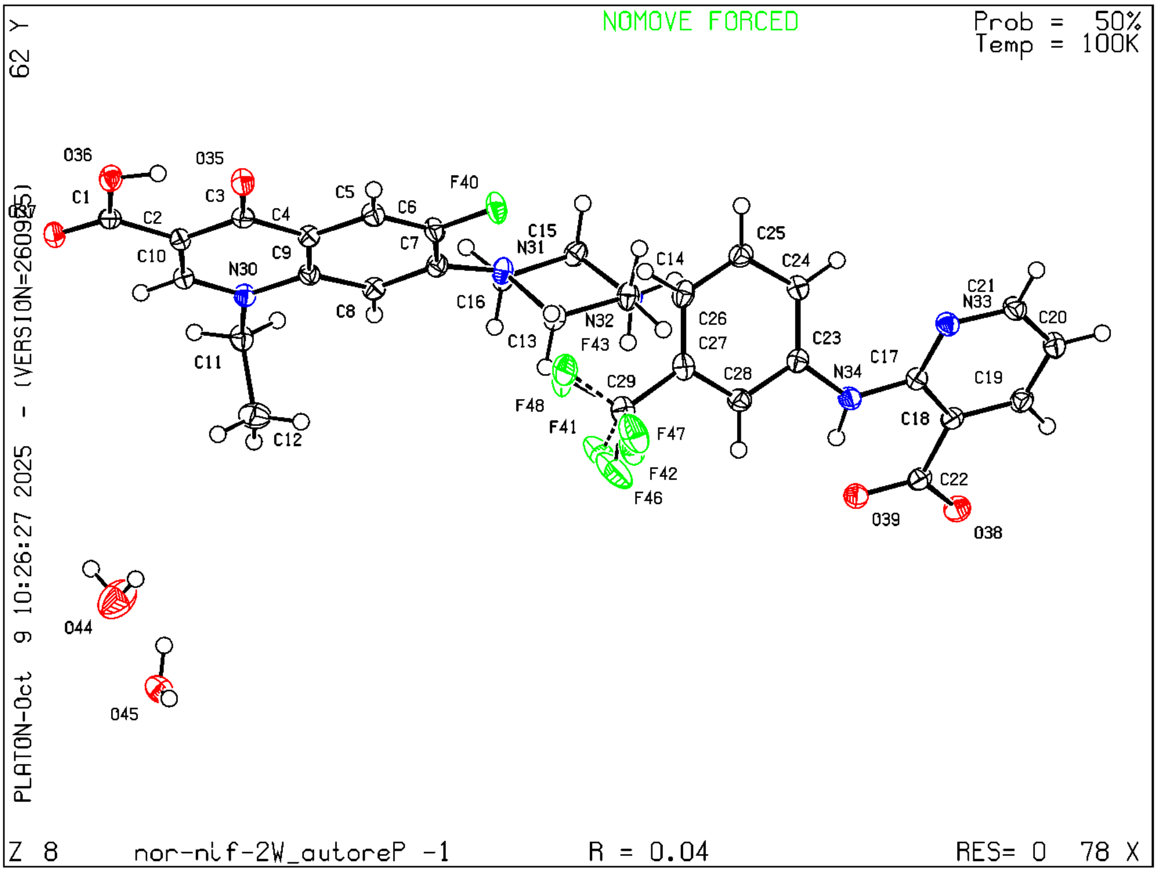

Supplement: Supplementary file 1 [file molecules-31-03189-s001.zip › Supplementary File S1 checkcif NORNIF.pdf]
