# Supplementary material for: A Comprehensive Study of a New Norfloxacin-Niflumate Hydrate: Structural and Physicochemical Properties, Antibiotic Potency, Anti-Inflammatory Effect, and Drug Safety
Source: Molecules. 2026 Sep 10;31(18):3189. doi: 10.3390/molecules31183189 (PMC13635407; doi:10.3390/molecules31183189)
Supplement: Supplementary file 1 [file molecules-31-03189-s001.zip › Supplementary File S2 NORNIF.pdf]

Supplementary File S2

Chromatogram and HPLC validation parameters

1. Chromatogram of NOR

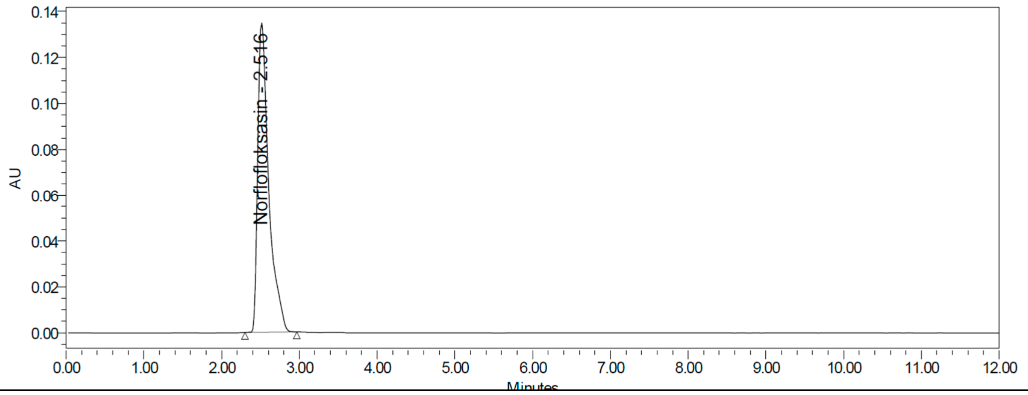

2. Chromatogram of NIF

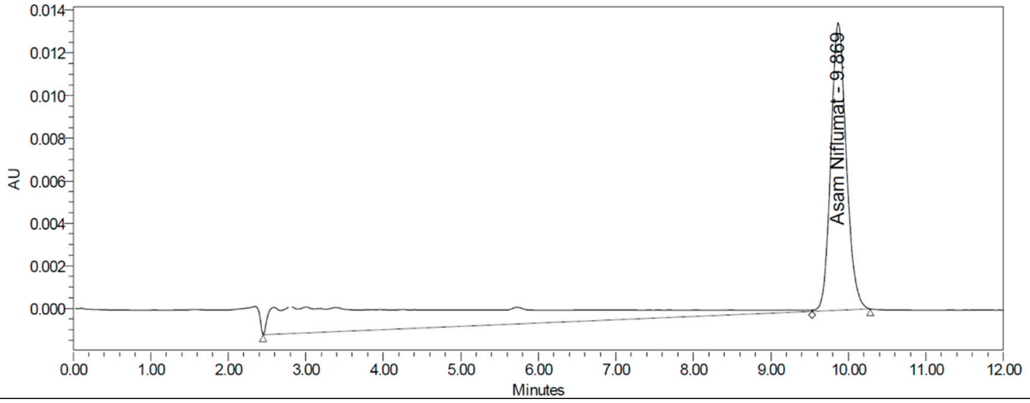

3. Chromatogram of NORNIF

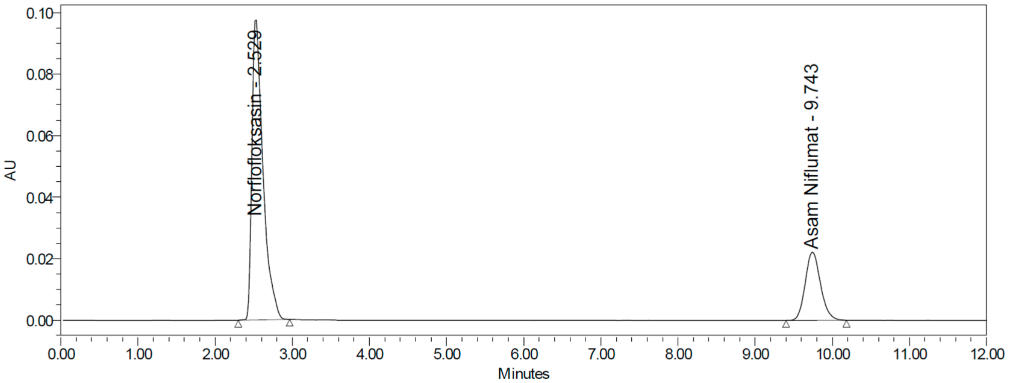

HPLC System Suitability Test

Parameters	Result	References
Tailing factors	1.502 ± 0.395	<2.0
Theoretical plates	11878.65 ± 47.34	N>2000
Capacity factors	8.891 ± 0.008	k>2
Resolution	2.505 ± 0.007	Rs > 1.5

Validation parameters

Parameters	Result	References
Linearity (R^2)	0.9985 ± 0.0006	>0.999
(%) RSD area under the curve (AUC)	0.200 ± 0.031	≤2%
(%) RSD of retention time	0.205 ± 0.139	≤1%
% recovery	101.7% ± 2.7	98 – 102%

Stability Data of NOR

Sample	AUC	Sample weight	Conc NOR (ug/mL)	Dilution Factor	Conc NOR compared to standard (%)
NOR standard 430 ug/mL	677569				
NOR week 4	591753	4.1 mg	352	10	85.84%
NOR week 4	591941	4.1 mg	352.1	10	85.87%
NOR week 4	591990	4.1 mg	352.1	10	85.88%
				AVERAGE	85.86%
